# A duality based 2-approximation algorithm for maximum agreement forest

**DOI:** 10.1007/s10107-022-01790-y

**Published:** 2022-03-21

**Authors:** Neil Olver, Frans Schalekamp, Suzanne van der Ster, Leen Stougie, Anke van Zuylen

**Affiliations:** 1 Department of Mathematics, London School of Economics and Political Science, London, United Kingdom; 2 Centrum Wiskunde & Informatica, Amsterdam, Netherlands; 3 School of Operations Research and Information Engineering, Cornell University, Ithaca, USA; 4 Department of Computer Science, Cornell University, Ithaca, USA; 5 Albert Heijn Online, Zaandam, Netherlands; 6 Department of Operations Analytics, Vrije Universiteit Amsterdam, Amsterdam, Netherlands; 7 INRIA-Erable, Lyon, France

**Keywords:** Maximum agreement forest, Phylogenetic tree, SPR distance, Subtree prune-and-regraft distance, Computational biology, 68W25, 90C27, 92D15

## Abstract

We give a 2-approximation algorithm for the Maximum Agreement Forest problem on two rooted binary trees. This NP-hard problem has been studied extensively in the past two decades, since it can be used to compute the rooted Subtree Prune-and-Regraft (rSPR) distance between two phylogenetic trees. Our algorithm is combinatorial and its running time is quadratic in the input size. To prove the approximation guarantee, we construct a feasible dual solution for a novel exponential-size linear programming formulation. In addition, we show this linear program has a smaller integrality gap than previously known formulations, and we give an equivalent compact formulation, showing that it can be solved in polynomial time.

## Introduction

Evolutionary relationships are often modeled by a rooted tree, where the leaves represent a set of species, and internal nodes are (putative) common ancestors of the leaves below the internal node. Such phylogenetic trees date back to Darwin [11], who used them in his notebook to elucidate his thoughts on evolution. For an introduction to phylogenetic trees we refer to [12, 24].

The topology of phylogenetic trees can be based on different sources of data, e.g., morphological data, behavioral data, genetic data, etc., which can lead to different phylogenetic trees on the same set of species. Such partly incompatible trees may actually be unavoidable: there exist non-tree-like evolutionary processes that preclude the existence of a phylogenetic tree, so-called reticulation events, such as hybridization, recombination and horizontal gene transfer [17, 18]. Irrespective of the cause of the conflict, the natural question arises to quantify the dissimilarity between such trees. Especially in the context of reticulation, a particularly meaningful measure of comparing phylogenetic trees is the Subtree Prune-and-Regraft distance for rooted trees (rSPR-distance), which provides a lower bound on a certain type of these non-tree evolutionary events. The problem of finding the exact value of this measure for a set of species motivated the formulation of the Maximum Agreement Forest Problem (MAF) by Hein, Jian, Wang and Zhang [16].

In the definition of MAF by Hein et al. we are given two rooted binary trees and a bijection from the leaves of each tree to a given set of labels ${{{\mathcal {L}}}}$. The problem is to find a minimum set of edges to be deleted from the two trees, so that the rooted trees in the resulting two forests form *isomorphic* pairs. Here, and throughout the paper, two rooted trees are said to be isomorphic if (i) the labelled nodes of the two trees have the same subset of labels, say *A*, and (ii) the two trees give rise to the same tree if we take the minimal subtree spanning the nodes labelled by *A* and repeatedly identify a node with its child if it only has a single child.

Since the introduction by Hein et al. in [16], in which they also proved NP-hardness, MAF has been extensively studied, mostly in its version of two rooted binary input trees. After Allen and Steel [1] pointed out that the claim by Hein et al. that solving MAF on two rooted directed trees computes the rSPR-distance between the trees is incorrect, Bordewich and Semple [5] presented a subtle redefinition of MAF, whose optimal value does coincide with the rSPR-distance. In this redefinition, the set of labels is extended with a label $\rho$, which is assigned to the roots of the two input trees. As before, we want to find a minimum set of edges so that the trees in the resulting forests form isomorphic pairs; note that the fact that the roots of the input trees have labels means that now there must be an isomorphic pair of trees in the resulting forests containing the (original) roots. This has now become the standard definition of MAF, for which Bordewich and Semple [5] showed that NP-hardness still holds, and Rodrigues [20] showed that it is in fact APX-hard.

The problem has attracted a lot of attention, and indeed has become a canonical problem in the field of phylogenetic networks. Many variants of MAF have been studied, including versions where the input consists of more than two trees [6, 7], and where the input trees are unrooted [29, 30] or non-binary [22, 27]. We will concentrate on MAF in its classical form with two rooted binary input trees, and we will be concerned with the worst-case approximability of the problem. The literature includes many other approaches to the problem, including fixed-parameter tractable algorithms (e.g., [28, 30]) and integer linear programming [31, 32]. But the quest for better approximation algorithms has become central within the MAF literature.

The first approximation algorithm for the problem with a fully correct analysis was given by Bonet et al. [3] in 2006; they obtain an approximation factor of 5, with a running time that is linear in the number of leaves. (The algorithm follows closely the approach taken by Hein et al. [16] and Rodrigues et al. [21], who both claimed 3-approximation algorithms; but both papers turned out to have flaws in the analysis.) This was followed by a sequence of three papers, each obtaining a 3-approximation algorithm. The first, by Bordewich et al. [4], had a running time of $O(n^5)$, where *n* denotes the number of leaves; Rodrigues et al. [22] substantially improved the running time to $O(n^2)$. Finally, Whidden and Zeh [28] simplified the analysis and improved the running time to *O*(*n*), matching the running time of the previous 5-approximation.

These algorithms all take a similar approach, and make decisions that are in a certain sense based on “local” information. We focus here on the algorithm and analysis of Whidden and Zeh [28] (based on [22]), since it is the cleanest. The algorithm maintains a tree $T_1'$ and a forest $T_2'$; initially, these are precisely the two input trees. $T_1'$ and $T_2'$ always have the same leaf set, which shrinks as the algorithm progresses; a leaf is removed when the part of the algorithm’s solution involving that leaf has been determined. The algorithm proceeds by considering any pair of leaves *a*, *b* in $T_1'$ that are siblings (two nodes are *siblings* in a tree if they have the same parent). Consider their situation in $T_2'$. If they are also siblings in $T_2'$, then there is clearly no reason to separate *a* and *b* in a solution, and they can be contracted together in both $T_1'$ and $T_2'$ to yield a smaller instance. Otherwise, the algorithm deletes the edges directly above *a* and *b* in both $T_1'$ and $T_2'$, resulting in two “trivial” trees consisting of a single leaf each that can essentially be removed from the instance; and also makes one further cut in $T_2'$, which will be the edge directly above a sibling of either *a* or *b* in $T_2'$. The process of merging and deleting edges is then continued on the new instance, until eventually a valid solution is found. (Note that the algorithm might at first glance appear to create many trivial trees consisting of only a single leaf; however, single leaves later in the algorithm may represent larger collections of leaves that have been merged together in earlier iterations.) A fairly direct combinatorial charging argument is used to show that in each iteration of the algorithm (where the algorithm makes three cuts), at least one edge deleted in the optimal solution can be uniquely charged for this iteration.

The next improvement in approximation factor, to a 2.5-approximation (at the cost of an increased quadratic running time) came from Shi et al. [25]. Their approach, like the 3-approximation algorithm described above, starts by choosing a pair of leaves *a*, *b* that are siblings in the first tree. However, it pays more attention to the configuration of the second tree and the positioning of *a* and *b* within it when deciding what edges to cut. Since larger structures are considered, the analysis is substantially more involved. A further improvement to a factor of 7/3 was then obtained by Chen, Machida and Wang [10]; their algorithm also runs in quadratic time. Again, larger combinatorial structures play a role; further, it does not begin with an arbitrary pair of sibling leaves in the first tree, but chooses the pair more carefully.

The first 2-approximation algorithm was given by a subset of the authors of the current work [23] (independently and essentially concurrently with the 7/3-approximation algorithm of Chen et al. [10]). They do not explicitly discuss (or attempt to optimize) the running time of the algorithm, beyond showing that it is polynomial time. Subsequently, Chen, Harada and Wang [8] (see also [9]), building on the 7/3-approximation algorithm [10], gave a very different factor 2 approximation algorithm, with a cubic running time.

The 2-approximation algorithm presented in the current paper may be viewed as the full version of the algorithm in [23]. However, while the algorithm presented here is similar in spirit, it differs in many details, and the exposition is entirely new. Although the algorithm and analysis remain quite subtle, this version is significantly shorter and clearer. Moreover, we show how our algorithm can, with some care, be implemented in quadratic time ( [23] discusses only a polynomial time bound). This improves over the cubic running time of Chen et al. [8].

Our 2-approximation algorithm differs from previous works in two key aspects.

- Our algorithm takes a global approach; choices made by the algorithm may depend on large parts of the instance. This is in contrast to the “local” algorithms discussed above. The cubic 2-approximation by Chen et al. [8] also requires non-local substructures, suggesting this may be a crucial factor in achieving this approximation bound.
- We introduce a novel integer linear programming formulation for the analysis. Our approximation guarantee is proved by constructing a feasible solution to the dual of this linear program, rather than arguing locally about the objective of the optimal solution. We thus bring a powerful tool from the theory of approximation algorithms to bear, one that has not been exploited in the study of MAF so far.
  We use the integer linear programming formulation, and in particular, its linear relaxation, only in our analysis. The algorithm itself is purely combinatorial. It is essentially a dual-fitting algorithm: the analysis explicitly constructs a dual solution with objective value at least half the cost of the primal solution returned by the algorithm.

Although we do not need to solve the linear programming (LP) relaxation, it is an interesting object of study, and it is natural to ask if it can indeed be efficiently optimized. This is not immediately clear, since the formulation has an exponential number of variables. Being able to solve the LP may, for example, be of future utility in obtaining better approximation guarantees using LP-rounding techniques. We show that the relaxation can be reformulated as a compact LP, with only a polynomial number of variables and constraints. This immediately implies that it can be optimized efficiently (in polynomial time). This may make the integer linear program amenable for use with commercial integer programming solvers. There is a previous formulation due to Wu [31], but our formulation is significantly stronger: the integrality gap of the relaxation of Wu is at least 3.2, whereas for ours we show it is at most 2, and in fact the worst example that we are aware of has integrality gap 1.25 (see the Appendix).

We have implemented and tested our algorithm, as well as the compact formulation [19]. The implementation has been designed so that it is easy to step through the algorithm and explore its behaviour on a given instance; the reader may find it helpful when examining the technical details of the algorithm.

*Outline* We define the problem and introduce necessary notation in Sect. 2. Section 3 describes the algorithm, and proves that it produces a feasible solution to MAF. In Sect. 4, we introduce the linear program, and describe a feasible solution to its dual that can be maintained by the algorithm. We then show the objective value of this dual solution is always at least half the objective value of the MAF solution, which proves the approximation ratio of 2. In Sect. 5, we show a compact formulation of the (exponential sized) linear program used for the analysis. Section 6 gives some concluding remarks and directions for further research. Finally, in the appendices, we provide the details on how to implement our algorithm so that it runs in time quadratic in the size of the input, and we give an example that shows that a previously known integer linear program [31] is not as strong as the formulation introduced here.

## Preliminaries

The input to the Maximum Agreement Forest problem (MAF) consists of two rooted binary trees $T_1$ and $T_2$. There is a bijection from the leaves of each tree to a given set of labels ${{{\mathcal {L}}}}$.

Let $V_1$ and $V_2$ denote the node sets of $T_1$ and $T_2$ respectively, and let $V = V_1 \cup V_2$. We will take a small liberty, and treat ${{{\mathcal {L}}}}$ as being a subset of $V_1$ and a subset of $V_2$. We call all nodes in $V \setminus {{{\mathcal {L}}}}$ *internal nodes*. We let ${{{\mathcal {L}}}}(u)$ denote the set of leaves that are descendants of a node $u \in V$.

We will use the following notational conventions: we use *u* and *v* to denote arbitrary nodes (including leaves); if the node we refer to is an internal node in $V_2$, we will use $\hat{u}$ and $\hat{v}$; and we use the letters *x*, *y* and *w* to refer to leaves.

For $A\subset {{{\mathcal {L}}}}$ we use $V_i[A]$ to denote the set of nodes in $T_i$ that lie on a path between any two leaves in *A* for $i \in \{1,2\}$, and define $V[A] := V_1[A] \cup V_2[A]$.

### Definition 1

We say that a set $A \subseteq {{{\mathcal {L}}}}$ *covers* a node $u \in V$ if $u \in V[A]$. We say that $A, A' \subseteq {{{\mathcal {L}}}}$ *overlap* if $V[A] \cap V[A'] \ne \emptyset$; we also say that *A* *overlaps* $A'$ in *U*, for $U \subseteq V$, if $V[A] \cap V[A'] \cap U \ne \emptyset$. We say a partition ${\mathcal {P}}$ of ${{{\mathcal {L}}}}$ *overlaps* in $U \subseteq V$ if there exist $A,A'\in {\mathcal {P}}$, $A\ne A'$, such that *A* and $A'$ overlap in *U*.

To give some intuition for the use of this definition, recall from the introduction that the goal of the MAF problem is to find a minimum set of edges to be deleted from the two input trees, so that the trees in the resulting two forests can be matched up into isomorphic pairs. One of the requirements for a pair of trees to be isomorphic is that they have the same set of labelled nodes. In other words, the trees in the two forests induce the same partition ${\mathcal {P}}$ of ${{{\mathcal {L}}}}$, and the fact that the forests are formed by deleting edges from the input trees means that no two sets in ${\mathcal {P}}$ overlap.

Next, we will give a definition that allows us to precisely express the other requirement for a pair of trees to be isomorphic. For $A\subseteq {{{\mathcal {L}}}}$, we let ${{\,\mathrm{lca}\,}}_i(A)$ denote the lowest common ancestor of *A* in $T_i$. We will sometimes omit braces of explicit sets and write, e.g., ${{\,\mathrm{lca}\,}}_1(x_1,x_2,x_3)$ instead of ${{\,\mathrm{lca}\,}}_1(\{x_1,x_2,x_3\})$. For nodes *u*, *v* in the same tree, we use $u\prec v$ to indicate that *u* is a descendant of *v* and $u \preceq v$ if *u* is equal to *v* or a descendant of *v*.

### Definition 2

A set $L\subseteq {{{\mathcal {L}}}}$ is *compatible * if for all $x_1,x_2,x_3\in L$

$$\begin{aligned} {{\,\mathrm{lca}\,}}_1(x_1,x_2)\prec {{\,\mathrm{lca}\,}}_1(x_1,x_2,x_3) \Leftrightarrow {{\,\mathrm{lca}\,}}_2(x_1,x_2)\prec {{\,\mathrm{lca}\,}}_2(x_1,x_2,x_3). \end{aligned}$$

We call a set of leaves *incompatible * if it is not a compatible set. Note that $L \subseteq {{{\mathcal {L}}}}$ is compatible precisely if the minimum subtree spanning *L* in $T_1$ and the minimum subtree spanning *L* in $T_2$ are isomorphic.

A feasible solution to MAF is a partition ${\mathcal {P}}= \{ A_1, A_2, \ldots , A_k\}$ of ${{{\mathcal {L}}}}$ such that every component $A_i$ is compatible, and $A_i$ does not overlap $A_j$, for any $i \ne j$. The cost of this solution is defined to be $|{\mathcal {P}}|-1$. This cost corresponds to the number of edges that must be deleted from $T_1$, as well as the same number from $T_2$, so that in both of the resulting forests, each $A_i \in {\mathcal {P}}$ is the leaf set of a single tree.

### Remark

In order for MAF to correspond to the rSPR distance, it is necessary to add an additional label $\rho$ *to* ${{{\mathcal {L}}}}$ (see figure below), that is assigned to the roots of $T_1$ and $T_2$. This is the distinction between the original definition of MAF by Hein [16] and the correction by Bordewich and Semple [5]. To maintain the property that only leaves have labels, we instead add a new root to $T_1$ and $T_2$, which has as its two children a leaf labelled $\rho$ and the original root. We simply assume that this addition is already included in the input instance, after which there is no need to distinguish this additional leaf from the others.


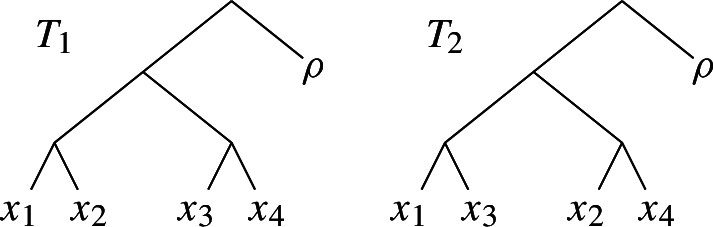


When we describe and analyze our algorithm, the following extended notion of compatibility is convenient.

### Definition 3

Given $K\subseteq {{{\mathcal {L}}}}$, we say a set $L\subseteq {{{\mathcal {L}}}}$ is *K-compatible* if $L\cap K$ is compatible. A partition ${\mathcal {P}}= \{ A_1, A_2, \ldots , A_k\}$ of ${{{\mathcal {L}}}}$ is *K-compatible* if $A_i$ is *K*-compatible for all $i=1,2,\ldots ,k$.

## The Red-Blue algorithm

The algorithm maintains a partition ${\mathcal {P}}$ of ${{{\mathcal {L}}}}$, which at the end of the algorithm will correspond to a feasible solution to MAF. The algorithm will maintain the invariant that ${\mathcal {P}}$ does not overlap in $V_2$. Observe that this is equivalent to defining ${\mathcal {P}}$ to be the leaf sets of the trees in a forest, obtained by deleting edges from $T_2$. Initially ${\mathcal {P}}= \{{{{\mathcal {L}}}}\}$.

Very informally, an iteration begins by coloring the leaves with three colors, red, blue, and white. The coloring is such that in $T_1$, there is a node *u* that has the red and blue leaves as its descendants; the set *B* of blue leaves is the set of “left” descendants of *u* and the set *R* of red leaves is the set of “right” descendants of *u*. The remaining leaves *W* are white. Furthermore, it will be the case that the current partition is feasible for the problem restricted to *R* and for the problem restricted to *B*. The current iteration will work to make the partition feasible for the problem restricted to $R\cup B$ (in fact, it will be feasible for the problem restricted to $R\cup B\cup \{w\}$ for all $w\in W$). Observe that a forest corresponding to a feasible solution to the full instance can have at most one tree that has leaves of multiple colors, because if there were two such trees then their leaf sets overlap on node *u* in $T_1$. Also, a multicolored tree in a feasible solution must be such that there is a node $\hat{u}$ in $T_2$ such that (i) no white leaf of the tree is a descendant of $\hat{u}$, and (ii) the blue and red leaves of the tree are left and right descendants of $\hat{u}$. We say the component is $(R\cup B)$-compatible if (ii) holds. The iteration will refine the multicolored components of the partition into (all but one) unicolored components. The natural idea would be to do this by intersecting each (or all but one) component with each color, but then the resulting partition might overlap in $V_2$; if not, we call the original partition splittable. So we first refine the partition such that it is splittable. In order to achieve the desired approximation guarantee, we need to be careful about the ordering of the steps we take to make the partition splittable , so that we can simultaneously maintain a feasible dual LP solution with an objective value that tracks the number of components; we do this by first making it $(R\cup B)$-compatible (which works toward splittability as well). Once the partition is $(R\cup B)$-compatible and splittable , we refine the partition by splitting all but at most one component into unicolored components. Finally, we look for a split that can be undone; the careful order in which the components are refined also serves to guarantee that such a merging of components is possible where needed to prove the approximation guarantee. We now give a precise definition, using the notation from the previous section.

As explained above, our algorithm works towards feasibility by iteratively refining ${\mathcal {P}}$, focusing each iteration on a set of leaves ${{{\mathcal {L}}}}(u)$ for some $u\in V_1$; *u* is a node such that the current partition is infeasible for ${{{\mathcal {L}}}}(u)$ in some (quite narrowly defined) way. At the end of the iteration the solution is feasible if we restrict our attention to ${{{\mathcal {L}}}}(u)$, and even if we consider ${{{\mathcal {L}}}}(u)\cup \{w\}$ for any arbitrary $w\in {{{\mathcal {L}}}}\setminus {{{\mathcal {L}}}}(u)$.

We use the following definition to specify which sets ${{{\mathcal {L}}}}(u)$ the algorithm considers.

### Definition 4

Given an infeasible partition ${\mathcal {P}}$ that does not overlap in $V_2$, we call $u\in V_1$ a *root of infeasibility* if at least one of the following holds:

- ${\mathcal {P}}$ is not ${{{\mathcal {L}}}}(u)$-compatible;
- $${\mathcal {P}}$$ overlaps in $$V_1[{{{\mathcal {L}}}}(u)]$$;
- ${\mathcal {P}}$ is ${{{\mathcal {L}}}}(u)$-compatible, and there exists a component $A\in {\mathcal {P}}$ such that $A\setminus {{{\mathcal {L}}}}(u)\ne \emptyset$ and $(A\cap {{{\mathcal {L}}}}(u))\cup \{w\}$ is incompatible for all $w\in A\setminus {{{\mathcal {L}}}}(u)$.

While the first two conditions can be naturally interpreted as failures of feasibility within $V_1[{{{\mathcal {L}}}}(u)]$, condition (c) is more subtle. It says that while *A* is ${{{\mathcal {L}}}}(u)$-compatible, *every* leaf $w \in A \setminus {{{\mathcal {L}}}}(u)$ provides a certificate that *A* is in fact incompatible. A different view of this is that every leaf in $A \setminus {{{\mathcal {L}}}}(u)$ lies below ${{\,\mathrm{lca}\,}}_2(A \cap {{{\mathcal {L}}}}(u))$ in $T_2$. We note that replacing condition (c) by requiring only the existence of at least one such leaf leads to an algorithm that appears to be “too greedy”; more precisely, the approximation guarantee we can prove in that case is worse than 2.

Observe that if $u\in V_1$ *is* a root of infeasibility , then any ancestor of *u* is a root of infeasibility as well. We will say an internal node *u* in tree $T_i$ is the “lowest” node with property $\Gamma$ if property $\Gamma$ does not hold for any of *u*’s descendants in $T_i$. The algorithm will thus identify a lowest node $u\in V_1$ that is a root of infeasibility.

**Fig. 1:**
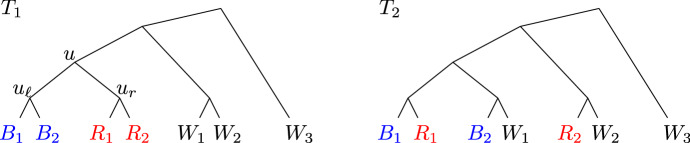
If ${\mathcal {P}}=\{{{{\mathcal {L}}}}\}$, then node *u* satisfies case (**a**) of Definition 4; if ${\mathcal {P}}= \{\{B_1\}$, $\{B_2,W_1\}$, $\{R_1, R_2, W_2, W_3\}\}$, it satisfies case (**b**) and if ${\mathcal {P}}=\{\{R_1\},$ $\{B_1, B_2, W_1, R_2, W_2\},$ $\{W_3\}\}$, it satisfies (**c**)

We illustrate the three conditions of a root of infeasibility in Fig. 1. $R_1$, $R_2$, $B_1$, $B_2$, $W_1$, $W_2$ and $W_3$ represent nonempty subtrees that appear in both $T_1$ and $T_2$ — for the examples it suffices to think of these as a subtree consisting of a single leaf. We will adopt this viewpoint and, with a slight abuse of notation, we will refer to the labels of these leaves as $R_1$, $R_2$, $B_1$, $B_2$, $W_1$, $W_2$ and $W_3$, respectively. If ${\mathcal {P}}=\{{{{\mathcal {L}}}}\}$, *u* satisfies (a). Note that *u* is indeed a lowest root of infeasibility, since $\{R_1,R_2,W_3\}$ and $\{B_1,B_2,W_3\}$ are compatible sets, so $u_\ell$ and $u_r$ do not satisfy (c) (nor (a) or (b)). If ${\mathcal {P}}= \{\{B_1\}, \{B_2, W_1\}, \{R_1, R_2, W_2, W_3\}\}$, node *u* satisfies (b). Again, *u* is a lowest root of infeasibility (clearly $u_\ell$ and $u_r$ do not satisfy (a) or (b); they also do not satisfy (c) since $\{B_2,W_1\}$ is compatible, as is $\{R_1,R_2,W_3\}$). Finally, if ${\mathcal {P}}=\{\{R_1\}, \{B_1, B_2, W_1, R_2, W_2\}, \{W_3\}\}$, node *u* satisfies (c). Observe that in this case *u* is again a lowest root of infeasibility. For $u' \in \{u_\ell , u_r\}$, (a) and (b) are clearly not satisfied; neither is (c) because the only $A\in {\mathcal {P}}$ such that $A\setminus {{{\mathcal {L}}}}(u')\ne \emptyset , A\cap {{{\mathcal {L}}}}(u')\ne \emptyset$ is $A=\{B_1,B_2, W_1,R_2,W_2\}$, but then $A\cap {{{\mathcal {L}}}}(u')\cup \{w\}$ is not incompatible for $w= W_2$ (and also not incompatible for $w=W_1$ if $u'=u_r$).

Given a root of infeasibility $u\in T_1$, we partition ${{{\mathcal {L}}}}$ into *R*, *B*, *W*, where $R={{{\mathcal {L}}}}(u_r)$ and $B={{{\mathcal {L}}}}(u_\ell )$ for the two children $u_r$ and $u_\ell$ of *u*. We will refer to this partition as a *coloring* of the leaves; we will refer to the leaves in *R* as red leaves, the leaves in *B* as blue leaves and the leaves in *W* as white leaves. We note that $u_r$ and $u_\ell$ are ${{\,\mathrm{lca}\,}}_1(R)$ and ${{\,\mathrm{lca}\,}}_1(B)$, respectively, and we use these interchangeably. We call a component of ${\mathcal {P}}$ *tricolored * if it has a nonempty intersection with *R*, *B* and *W*, and *bicolored * if it has a nonempty intersection with exactly two of the sets *R*, *B*, *W*. A component is called *multicolored * if it is either tricolored or bicolored , and *unicolored* otherwise.

### Observation 1

Let *u* be a lowest root of infeasibility for ${\mathcal {P}}$, and consider the coloring *R*, *B*, *W*, where $R={{{\mathcal {L}}}}(u_r)$ and $B={{{\mathcal {L}}}}(u_\ell )$ for the two children $u_r$ and $u_\ell$ of *u*. Then the set of multicolored components of ${\mathcal {P}}$ consists of either at most two bicolored components or exactly one tricolored component.

### Proof

If *u* is a lowest root of infeasibility, ${\mathcal {P}}$ does not overlap in $V_1[R]$ and $V_1[B]$, and so at most one component of ${\mathcal {P}}$ covers $u_r={{\,\mathrm{lca}\,}}_1(R)$, and at most one covers $u_\ell ={{\,\mathrm{lca}\,}}_1(B)$. Since any multicolored component covers at least one of ${{\,\mathrm{lca}\,}}_1(R)$ and ${{\,\mathrm{lca}\,}}_1(B)$, there can be at most two multicolored components. Furthermore, because any tricolored component covers both ${{\,\mathrm{lca}\,}}_1(R)$ and ${{\,\mathrm{lca}\,}}_1(B)$, if there is a tricolored component there can be no other multicolored component. $\square$

We note that the above observation can be refined; it is possible to show that ${\mathcal {P}}$ contains either one tricolored component or exactly two bicolored components; see Lemma 12 in Sect. 4.3.

We now give the overall algorithm. In the description, but also in the descriptions of the various procedures that follow, the $\star$ in front of certain lines will be used to refer to these lines in the analysis in Sect. 4.2.
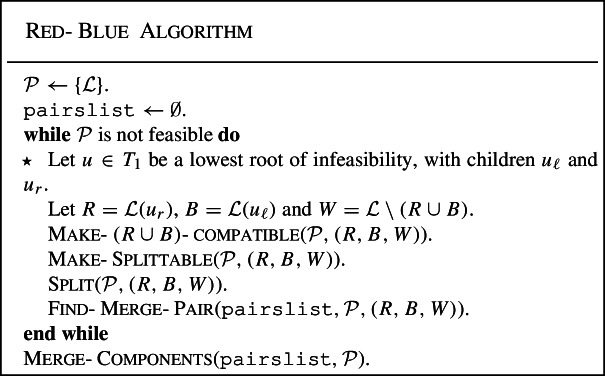


The various procedures in the Red-Blue Algorithm will be described in detail in the subsequent subsections, along with lemmas regarding the properties they ensure. For now, we give a very high-level description.

An iteration of the main while-loop starts by finding a lowest root of infeasibility *u*, yielding a coloring (*R*, *B*, *W*) of the vertices; if there is no root of infeasibility, then the current partition is feasible, and the main loop terminates. The goal of the iteration, essentially, is to ensure that by the end of the iteration, *u* is no longer a root of infeasibility, while maintaining the invariant that the partition does not overlap on $V_2$. Until the very end of the algorithm, the partition is only ever refined; since each iteration must modify the partition, the number of iterations is bounded by $|{{{\mathcal {L}}}}|$. (Alternatively, our analysis shows that if *u* is chosen for some iteration of the algorithm, then from the end of the iteration until the very end of the algorithm, *u* will never again be a root of infeasibility.)

The process of refining the partition to make *u* no longer a root of infeasibility proceeds in two main stages. First, the procedure Make-$(R\cup B)$-compatible refines the partition if necessary so that it is $(R\cup B)$-compatible, i.e., so that condition (a) fails to hold. The procedures Split and Make-Splittable will together ensure that conditions (b) and (c) also both fail to hold, so that *u* is no longer a root of infeasibility at the end of the iteration. In particular, they ensure that the partition does not overlap in $V_1[{{{\mathcal {L}}}}(u)]$, and that the final partition is $(R\cup B\cup \{w\})$-compatible for every $w \in {{{\mathcal {L}}}}$ (which is stronger than (c) not holding).

Finally, Find-Merge-Pairs and Merge-Components are needed for the approximation bound only. All the other steps in the algorithm only refine the current partition. In some particular cases, it is possible and necessary to undo some of these refinements. This is done in a careful way at the very end of the algorithm by Merge-Components, using information prepared by Find-Merge-Pairs. The reason that the merges are done at the end, rather than during the main loop, is primarily for analysis purposes.

In order to simplify the statement of the lemmas, we will make statements like “let ${\mathcal {P}}'$ be the partition after ProcedureName$({\mathcal {P}}, (R, B, W))$”. This implicitly assumes that (*R*, *B*, *W*) was a coloring chosen in the beginning of the current iteration of the Red-Blue Algorithm (and thus, that ${{\,\mathrm{lca}\,}}_1(R\cup B)$ was a lowest root of infeasibility at that moment), and that ${\mathcal {P}}'$ is the partition resulting from calling ProcedureName$({\mathcal {P}}, (R, B, W))$ in the current iteration.

### Make-$(R\cup B)$-compatible

If ${\mathcal {P}}$ is not $(R\cup B)$-compatible, we start by refining ${\mathcal {P}}$ with the following procedure so that each of its components is $(R\cup B)$-compatible.
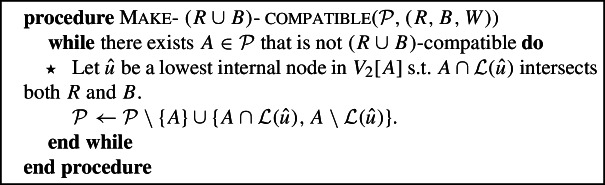

**Fig. 2:**
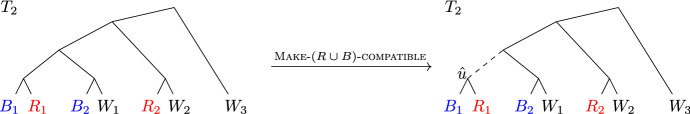
Illustration of Make-$(R\cup B)$ -compatible(${\mathcal {P}},(R,B,W)$). Because ${\mathcal {P}}$ and ${\mathcal {P}}'$ do not overlap in $V_2$, we can represent these partitions as the leaf sets of trees in a forest obtained by deleting edges from $T_2$. In this figure and the following figures the dashed edges represent deleted edges. In this example ${\mathcal {P}}=\{{{{\mathcal {L}}}}\}$. Then Make-$(R\cup B)$ -compatible(${\mathcal {P}},(R,B,W)$) must choose $\hat{u}={{\,\mathrm{lca}\,}}_2(R_1, B_1)$, and refines the partition to $\{\{B_1, R_1\}, \{B_2, W_1, R_2, W_2,W_3\}\}$, which is $(R\cup B)$-compatible

An example is given in Fig. 2. We note that in general, the choice of $\hat{u}$ does not have to be unique, and that multiple refinements may be needed to make the partition $(R\cup B)$-compatible .

As observed above, for any partition ${\mathcal {P}}$ that does not overlap in $V_2$, there is a set of edges in $T_2$ such that ${\mathcal {P}}$ consists of the leaf sets of the trees in the forest obtained after deleting these edges. Our refinement is equivalent to deleting the parent edge of $\hat{u}$, and hence the resulting partition does not overlap in $V_2$ if the original partition did not overlap in $V_2$.

#### Lemma 1

Let ${\mathcal {P}}'$ be the partition after Make-$(R\cup B)$ -compatible$({\mathcal {P}}, (R, B,W))$. Then ${\mathcal {P}}'$ is a refinement of ${\mathcal {P}}$ that does not overlap in $V_2$ and is $(R\cup B)$-compatible .

#### Proof

First, observe ${\mathcal {P}}$ is *R*-compatible and *B*-compatible, since *u*’s children are not roots of infeasibility. If ${\mathcal {P}}$ is $(R\cup B)$-compatible then ${\mathcal {P}}$ is not modified by the procedure, and the lemma is vacuously true. Otherwise, the procedure refines ${\mathcal {P}}$, and, as argued above, the resulting partition ${\mathcal {P}}'$ does not overlap in $V_2$ provided that ${\mathcal {P}}$ does not overlap in $V_2$. The procedure ends when there are no sets in ${\mathcal {P}}$ that are not $(R\cup B)$-compatible , so the only thing left to show is that this procedure halts. Because $\hat{u}$ was chosen to be the lowest internal node in $V_2[A]$ such that $A \cap {{{\mathcal {L}}}}(\hat{u})$ intersects both *R* and *B*, the children of $\hat{u}$, say $\hat{u}_r$ and $\hat{u}_\ell$, are so that $A \cap {{{\mathcal {L}}}}(\hat{u}_r)$ and $A \cap {{{\mathcal {L}}}}(\hat{u}_\ell )$ can only intersect one of *R* and *B*. Therefore $A\cap {{{\mathcal {L}}}}(\hat{u})$ is $(R\cup B)$-compatible , where *A* was not, and thus the number of $(R\cup B)$-compatible components in ${\mathcal {P}}$ increases, which can only happen at most $|{{{\mathcal {L}}}}|$ times. $\square$

Observe that if ${\mathcal {P}}$ is $(R\cup B)$-compatible , then any refinement of ${\mathcal {P}}$ is also $(R\cup B)$-compatible , and hence we may assume that the partition at any later point in the current iteration of the Red-Blue Algorithm is $(R\cup B)$-compatible .

### Make-splittable

The goal of the next two procedures is to further refine the partition so that there is no overlap in $V_1[R\cup B]$. We will do this in two steps. The first of these procedures will make the partition “splittable ”. To describe this informally, we view the components of the partition as the trees of the forest obtained by deleting edges from $T_2$. We call a component *A* that intersects *k* colors splittable , if there are $k-1$ edges that can be deleted from $T_2$ to “split” the tree into *k* unicolored components. We can phrase this property succinctly using the notion of overlapping: if the sets $A \cap R$, $A \cap B$ and $A \cap W$ do not overlap in $V_2$, then there are disjoint trees in $T_2$ that have each of these sets as leaf sets, and we can therefore split the tree associated with *A* in $T_2$ into these three trees by deleting at most two edges.

#### Definition 5

Given a coloring (*R*, *B*, *W*) of ${{{\mathcal {L}}}}$, a set $A \subseteq {{{\mathcal {L}}}}$ is *splittable* if $A \cap R$, $A\cap B$ and $A\cap W$ do not overlap in $V_2$. A partition is splittable if every component in the partition is splittable.


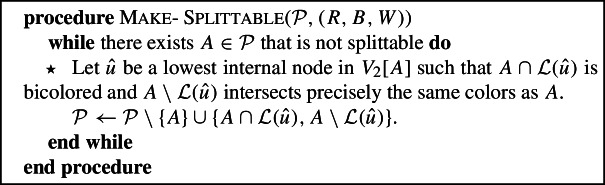


**Fig. 3:**
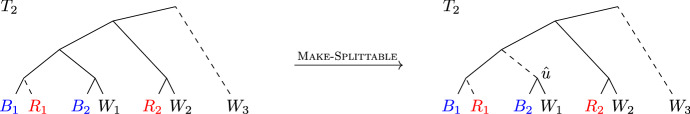
Illustration of Make-Splittable(${\mathcal {P}},(R,B,W)$). ${\mathcal {P}}=\{\{R_1\}, \{B_1, B_2, W_1, R_2, W_2\}, \{W_3\}\}$, and the set $A=\{B_1, B_2, W_1, R_2, W_2\}$ is not splittable . Make-Splittable(${\mathcal {P}}$) would choose $\hat{u}={{\,\mathrm{lca}\,}}_2(B_2, W_1)$ and replace *A* by $\{B_2,W_1\}$ and $\{B_1, R_2, W_2\}$

As a first example of Make-Splittable, consider ${\mathcal {P}}=\{\{B_1, R_1\}, \{B_2, W_1, R_2, W_2,W_3\}\}$ that was the output of Make-$(R\cup B)$ -compatible depicted in Fig. 2. In this example ${\mathcal {P}}$ is already splittable . In Fig. 3 a more interesting example is given.

#### Lemma 2

Make-Splittable is well-defined, in that a node $\hat{u}$ satisfying the desired properties in line $\star$ can always be found.

#### Proof

If *A* is bicolored and not splittable , then there exists $\hat{u}\in V_2[A]$ such that both $A\cap {{{\mathcal {L}}}}(\hat{u})$ and $A\setminus {{{\mathcal {L}}}}(\hat{u})$ are bicolored : just take $\hat{u}$ to be a lowest node in $V_2[A\cap C_1] \cap V_2[A\cap C_2]$ for distinct $C_1,C_2\in \{R, B, W\}$; such a node exists because *A* is not splittable, and the fact that $\hat{u}$ is in $V_2[A\cap C_i]$ for $i=1,2$ implies that $A\cap {{{\mathcal {L}}}}(\hat{u})$ and $A\setminus {{{\mathcal {L}}}}(\hat{u})$ intersect $C_i$.

It remains to prove the lemma for the case that *A* is tricolored . For this to hold, we need that ${\mathcal {P}}$ is $(R\cup B)$-compatible , which by Lemma 1 is indeed true when Make-Splittable is called. So suppose *A* is tricolored and not splittable . Note that $V_2[A\cap R]$ and $V_2[A\cap B]$ cannot intersect because *A* is $(R\cup B)$-compatible . Assume without loss of generality that $V_2[A\cap R]\cap V_2[A\cap W]\ne \emptyset$, and let $\hat{u}$ be a lowest node in $V_2[A\cap R]\cap V_2[A\cap W]$. Note that both $A\cap {{{\mathcal {L}}}}(\hat{u})$ and $A\setminus {{{\mathcal {L}}}}(\hat{u})$ must intersect *W* and *R*, and that $A\cap {{{\mathcal {L}}}}(\hat{u})$ cannot intersect *B*, since then *A* would not be $(R\cup B)$-compatible . So $A\cap {{{\mathcal {L}}}}(\hat{u})$ is bicolored , and $A\setminus {{{\mathcal {L}}}}(\hat{u})$ is tricolored . $\square$

#### Lemma 3

Let ${\mathcal {P}}'$ be the partition after Make-Splittable$({\mathcal {P}}, (R, B,W))$. Then ${\mathcal {P}}'$ is a refinement of ${\mathcal {P}}$ that does not overlap in $V_2$ and in which every component is splittable .

#### Proof

By Lemma 2, and since each iteration increases the number of components in ${\mathcal {P}}$, Make-Splittable must terminate, and by its definition, the final partition ${\mathcal {P}}'$ contains only splittable components. Clearly ${\mathcal {P}}'$ is a refinement of ${\mathcal {P}}$; it does not overlap in $V_2$ by the same arguments as used in the proof of Lemma 1. $\square$

Before continuing, we summarize the properties of the partition resulting after Make-Splittable that will be useful in the proof of the approximation guarantee in Sect. 4. To describe these, we need the notion of a *top component*.

#### Definition 6

If *A* is a component in the partition at the beginning of an iteration, and *A* is multicolored , then *A* is a top component. If *A* is a top component of the current partition, and *A* gets subdivided into $A \setminus {{{\mathcal {L}}}}(\hat{u})$ and $A\cap {{{\mathcal {L}}}}(\hat{u})$ by Make-$(R\cup B)$ -compatible or Make-Splittable, then $A \setminus {{{\mathcal {L}}}}(\hat{u})$ (but not $A \cap {{{\mathcal {L}}}}(\hat{u})$) is a top component of the resulting partition.

We note that by Observation 1, there are always either exactly one or two top components at the start of the iteration, and hence throughout (until the call to Split, after which the notion is no longer defined).

#### Lemma 4

Let ${\mathcal {P}}^{(0)}$ denote the partition at the start of a given iteration, and (*R*, *B*, *W*) the coloring of the leaves that is selected, let ${\mathcal {P}}^{(1)}$ denote the partition after Make-$(R\cup B)$ -compatible(${\mathcal {P}}^{(0)}, (R, B, W)$), and let ${\mathcal {P}}^{(2)}$ denote the partition after Make-Splittable(${\mathcal {P}}^{(1)},(R, B, W)$). Then the following properties hold:

1. Only multicolored components are subdivided by the iteration, i.e., if $A\in {\mathcal {P}}^{(0)}\setminus {\mathcal {P}}^{(2)}$, then *A* is multicolored.
2. The number of tricolored components in ${\mathcal {P}}^{(2)}$ is the same as in ${\mathcal {P}}^{(1)}$.
3. Any tricolored component in ${\mathcal {P}}^{(1)}$ or ${\mathcal {P}}^{(2)}$ that is not a top component contains no compatible tricolored triple.
4. Any bicolored component *A* in ${\mathcal {P}}^{(2)}$ that is not a top component satisfies that ${{\,\mathrm{lca}\,}}_2(A)$ is not covered by $A\cap C$ for any color $C\in \{R, B, W\}$. In other words, ${{{\mathcal {L}}}}(\hat{u}_\ell ) \cap A$ and ${{{\mathcal {L}}}}(\hat{u}_r) \cap A$ are unicolored where $\hat{u}_\ell$ and $\hat{u}_r$ are the children of ${{\,\mathrm{lca}\,}}_2(A)$.
5. If $x_W$ is in component *A* in ${\mathcal {P}}^{(0)}$, and $x_W$ is not a descendant of ${{\,\mathrm{lca}\,}}_2(A\cap (R\cup B))$ (and thus $x_W$ is a white leaf) , then either $A\in {\mathcal {P}}^{(2)}$ or $x_W$ is in a top component in ${\mathcal {P}}^{(2)}$.

#### Proof

The fact that property 1 holds can be read from the description of Make-$(R\cup B)$ -compatible and Make-Splittable. Property 2 follows from the description of Make-Splittable.

For property 3, we prove that when a non-top component is created from a top component, this non-top component cannot have compatible tricolored triples. This implies that no non-top component can have a compatible tricolored triple. First consider non-top components created by Make-$(R\cup B)$ -compatible from a top component *A*. The fact that node $\hat{u}$ picked in Make-$(R\cup B)$ -compatible is always chosen as low as possible implies that when the non-top component $A'=A\cap {{{\mathcal {L}}}}(\hat{u})$ is created, it holds that ${{\,\mathrm{lca}\,}}_2(x_R,x_B) = \hat{u}$ for any $x_R \in A' \cap R, x_B \in A' \cap B$. Therefore, for any $x_W\in A'\cap W$, it must be the case that either ${{\,\mathrm{lca}\,}}_2(x_W,x_R)\prec \hat{u}$ or ${{\,\mathrm{lca}\,}}_2(x_W, x_B)\prec \hat{u}$. But then $\{x_R, x_B, x_W\}$ is incompatible , because ${{\,\mathrm{lca}\,}}_1(x_R,x_B) \prec {{\,\mathrm{lca}\,}}_1(x_R, x_B,x_W)$. So non-top components in ${\mathcal {P}}^{(1)}$ can indeed not have compatible tricolored triples. Non-top components created by Make-Splittable from a top component are bicolored by definition, so these cannot have compatible tricolored triples either. Therefore, property 3 holds.

A similar argument shows property 4. First, consider a non-top component *A* created by Make-$(R\cup B)$ -compatible. *A* intersects *R* and *B*, so if *A* is bicolored , it contains no white leaves, so ${{\,\mathrm{lca}\,}}_2(A)$ is not covered by $A\cap W=\emptyset$. Now, because ${{\,\mathrm{lca}\,}}_2(A)$ is the node $\hat{u}$ picked in Make-$(R\cup B)$ -compatible, which is as low as possible, ${{\,\mathrm{lca}\,}}_2(A)$ is not covered by $A\cap R$ nor $A\cap B$. For a non-top component *A* created by Make-Splittable, the fact that ${{\,\mathrm{lca}\,}}_2(A)$ is the node $\hat{u}$ picked in Make-Splittable which is chosen as low as possible again implies that ${{\,\mathrm{lca}\,}}_2(A)$ is not covered by $A\cap C$ for any color $C\in \{R, B, W\}$.

For property 5, if $A\not \in {\mathcal {P}}^{(2)}$, consider a node $\hat{u}$ selected by Make-$(R\cup B)$ -compatible or Make-Splittable that leads to a subdivision of *A*. It suffices to argue that $\hat{u}\preceq {{\,\mathrm{lca}\,}}_2(A\cap (R\cup B))$, because then the fact that $x_W$ is not a descendant of ${{\,\mathrm{lca}\,}}_2(A\cap (R\cup B))$ implies that $x_W$ always remains in a top component. For $\hat{u}$ selected by Make-$(R\cup B)$ -compatible this fact holds because $\hat{u}$ is a lowest node such that $A\cap {{{\mathcal {L}}}}(\hat{u})$ intersects *R* and *B*. For $\hat{u}$ selected by Make-Splittable this fact holds because $\hat{u}$ is a lowest node such that $A\cap {{{\mathcal {L}}}}(\hat{u})$ is bicolored , and $A\setminus {{{\mathcal {L}}}}(\hat{u})$ intersects the same colors as *A*. $\square$

### Split

We now “split” the multicolored components of the partition: essentially, we further refine the partition by intersecting each multicolored component with *R*, *B* and *W*. Thus a component intersecting *k* colors will be split into *k* unicolored components. The fact that the components of the partition were splittable ensures that the resulting partition does not overlap in $V_2$. We will, however, need to be slightly more careful in order to achieve the approximation guarantee; in particular, we will sometimes need to perform what we call a Special-Split.
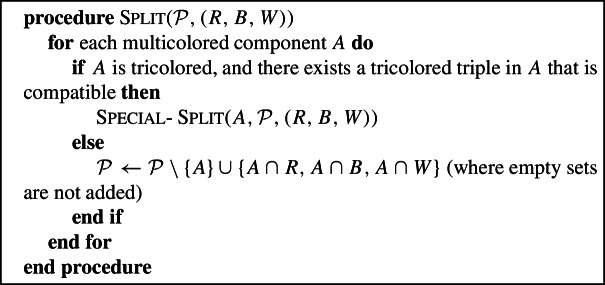


#### Remark

Our analysis in Sect. 4 needs the Special-Split, Find-Merge-Pair and Merge-Components procedures only in one (of three) cases that will be described in Lemma 12. Without these procedures, it is trivial to see that the resulting partition is feasible, and we will see in Sect. 4 that the proof of the approximation ratio is quite simple in these cases. On first reading, the reader may thus choose to skip the description of these procedures, and also read Sect. 4 only up to the proof of Proposition 14.

We emphasize that the Special-Split procedure is only called if *A* is tricolored , and there is at least one tricolored compatible triple in *A*. Hence, by property 3 of Lemma 4, Special-Split is only applied to tricolored top components.
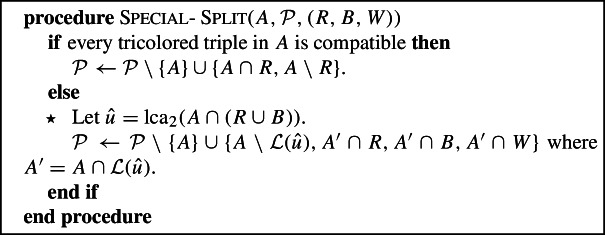


**Fig. 4:**
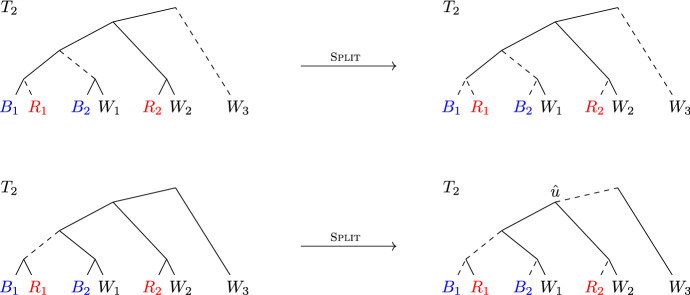
Two illustrations of Split(${\mathcal {P}},(R,B,W)$). In the top example ${\mathcal {P}}=\{\{R_1\},$ $\{B_2,W_1\},$ $\{B_1,R_2,W_2\},$ $\{W_3\}\}$ and Split(${\mathcal {P}}$) would simply refine each set of ${\mathcal {P}}$ by intersecting it with the three color classes. The result is that every leaf is a singleton in ${\mathcal {P}}'$. In the bottom example, ${\mathcal {P}}=\{\{B_1,R_1\}, \{B_2, W_1, R_2, W_2, W_3\}\}$. The set $A=\{B_2, W_1, R_2, W_2, W_3\}$ is tricolored and contains triple $\{B_2, R_2, W_3\}$ that is tricolored and compatible , but not every tricolored triple in *A* is compatible , e.g., $\{B_2,R_2, W_2\}$ is not compatible . In this case, the Special-Split replaces *A* by $\{\{B_2\}, \{R_2\},$ $\{W_1,W_2\},$ $\{W_3\}\}$

We refer to Fig. 4 for examples of the split operations in the two cases.

We now describe the property that the partition produced by Split will have, which goes beyond merely being $(R\cup B)$-compatible and non-overlapping in $V_2$ and $V_1[R\cup B]$.

#### Definition 7

Let $K \subseteq {{{\mathcal {L}}}}$. A partition ${\mathcal {P}}$ is *K-feasible* if for all $w\in {{{\mathcal {L}}}}$, ${\mathcal {P}}$ is $K\cup \{w\}$-compatible, and no two components in ${\mathcal {P}}$ overlap in $V_2\cup V_1[K]$.

**Fig. 5:**
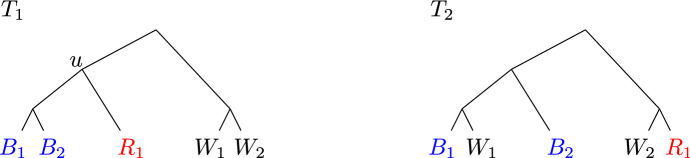
An example where ${\mathcal {P}}$ is $(R\cup B)$-compatible and does not overlap in $V_2\cup V_1[R\cup B]$, but that is not $(R\cup B$)-feasible. In this example, ${\mathcal {P}}=\{{{{\mathcal {L}}}}\}$, which clearly does not overlap in any node. If we stop the current iteration with ${\mathcal {P}}$, then ${{\,\mathrm{lca}\,}}_1(\{B_1,B_2\})$ and ${{\,\mathrm{lca}\,}}_1(\{W_1, W_2\})$ are lowest roots of infeasibility; no matter which one is chosen, the next iteration would further subdivide the partition restricted to $R\cup B$. Because we want to ensure this does not happen, the current iteration of the Red-Blue Algorithm will further subdivide the partition induced on $R\cup B$: it will create components $\{B_1,W_1\}, \{B_2, W_2, R_1\}$ in Make-Splittable and split everything into singleton components in Split

**Fig. 6:**
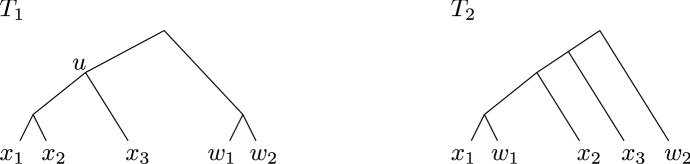
An example where ${\mathcal {P}}$ is not ${{{\mathcal {L}}}}(u)$-feasible, but *u* is not a root of infeasibility. (To emphasize that *u* is not a root of infeasibility, the leaves are labelled with $x_1$, $x_2$, $x_3$, $w_1$ and $w_2$, in contrast to earlier figures.) In this example, ${\mathcal {P}}=\{{{{\mathcal {L}}}}\}$, which does not overlap in any node, and ${\mathcal {P}}$ is ${{{\mathcal {L}}}}(u)$–compatible because the triple ${{{\mathcal {L}}}}(u)$ is compatible. But ${\mathcal {P}}$ is not ${{{\mathcal {L}}}}(u)$-feasible because ${{{\mathcal {L}}}}(u)\cup \{w_1\}$ is not compatible . On the other hand, *u* is not a root of infeasibility because ${{{\mathcal {L}}}}(u)\cup \{w_2\}$ is compatible

We will simply say ${\mathcal {P}}$ is *feasible* if it is ${{{\mathcal {L}}}}$-feasible, which we note does indeed coincide with the definition of a feasible solution to MAF. We make two additional remarks about the notion of *K*-feasibility:

- This stronger compatibility notion will be used in Lemma 7 to show that if ${\mathcal {P}}$ is $(R\cup B)$-feasible, then future iterations of the Red-Blue Algorithm will not further subdivide (the restriction of the partition to) $R\cup B$. This is not necessarily true if ${\mathcal {P}}$ is only $(R\cup B)$-compatible and does not overlap in $V_2\cup V_1[R\cup B]$. See Fig. 5 for an example.
- If $u\in V_1$ is a root of infeasibility for ${\mathcal {P}}$, then ${\mathcal {P}}$ is not ${{{\mathcal {L}}}}(u)$-feasible. The converse is not true, however: if ${\mathcal {P}}$ contains a single component containing ${{{\mathcal {L}}}}(u)$ which is ${{{\mathcal {L}}}}(u)$-compatible, but this component contains both $w\in {{{\mathcal {L}}}}\setminus {{{\mathcal {L}}}}(u)$ such that ${{{\mathcal {L}}}}(u)\cup \{w\}$ is compatible, and $w'\in {{{\mathcal {L}}}}\setminus {{{\mathcal {L}}}}(u)$ such that ${{{\mathcal {L}}}}(u)\cup \{w'\}$ is not compatible, then ${\mathcal {P}}$ is *not* ${{{\mathcal {L}}}}(u)$-feasible, but *u* is not a root of infeasibility. See Fig. 6 for an example. The stronger notion of a *u* being a root of infeasibility versus not being ${{{\mathcal {L}}}}(u)$-feasible is needed when we prove the approximation guarantee in Sect. 4.

Before we prove that the outcome of Split is $(R\cup B)$-feasible, we prove the following technical lemma that gives sufficient conditions for a partition to not overlap in $V_1[R\cup B]$.

#### Lemma 5

Let ${\mathcal {P}}$ be the partition and (*R*, *B*, *W*) be the coloring at the start of an iteration. Let ${\mathcal {P}}'$ be a refinement of ${\mathcal {P}}$ that does not overlap in $V_2$ and that is $(R\cup B)$-compatible . Then ${\mathcal {P}}'$ does not overlap in $V_1[R\cup B]$ if the following two conditions are met:

(i) ${\mathcal {P}}'$ has at most one multicolored component;
(ii) for the multicolored component $A^*\in {\mathcal {P}}'$ (if it exists), either ${{\,\mathrm{lca}\,}}_2(R\cup B) \prec {{\,\mathrm{lca}\,}}_2(A^*)$ or any node $\hat{v}$ with ${{\,\mathrm{lca}\,}}_2(A^*) \prec \hat{v} \preceq {{\,\mathrm{lca}\,}}_2(R\cup B)$ is covered only by components in ${\mathcal {P}}'$ that are subsets of *W*, or that are also components of ${\mathcal {P}}$.

#### Proof

Suppose the conditions of the lemma hold for ${\mathcal {P}}'$. First, observe that ${\mathcal {P}}'$ having at most one multicolored component implies that ${\mathcal {P}}'$ contains at most one component covering ${{\,\mathrm{lca}\,}}_1(R\cup B)$. Hence, if we suppose for a contradiction $A',A''\in {\mathcal {P}}'$ exist that overlap in $V_1[R\cup B]$, then they must overlap in $V_1[R]$ or $V_1[B]$. Without loss of generality, assume that $A', A''\in {\mathcal {P}}'$ overlap in $V_1[R]$. Since they do not overlap in ${{\,\mathrm{lca}\,}}_1[R\cup B]$, we may assume also without loss of generality that $A'\subseteq R$ and $A''\subseteq R\cup B\cup W$.

Since ${{\,\mathrm{lca}\,}}_1(R\cup B)$ was chosen as a lowest root of infeasibility, ${{\,\mathrm{lca}\,}}_1(R)$ was not a root of infeasibility for ${\mathcal {P}}$. This implies that no two components of ${\mathcal {P}}$ overlap in $V_1[R]$, so it must be the case that $A'$ and $A''$ were both part of a single component in ${\mathcal {P}}$ and were split. Also, ${\mathcal {P}}$ must have been *R*-compatible, so $(A'\cup A'')\cap R$ is a compatible set. We will show that these facts imply that if $A'$ and $A''$ overlap in $V_1[R]$, then they must overlap in $V_2[R]$, thus contradicting that ${\mathcal {P}}'$ does not overlap in $V_2$.

Let *v* be a lowest node in $V_1[R]$ such that $A'\cap {{{\mathcal {L}}}}(v) \ne \emptyset$ and $A''\cap {{{\mathcal {L}}}}(v)\ne \emptyset$ where we note that *v* exists since $A',A''$ overlap in some node in $V_1[R]$. Observe that a child of *v* cannot be in both $V_1[A']$ and $V_1[A'']$, as this contradicts the choice of *v*, and *v* itself is in $V_1[A']$ and $V_1[A'']$ only if $A'$ and $A''$ also contain leaves in ${{{\mathcal {L}}}}\setminus {{{\mathcal {L}}}}(v)$. Let $x',x''$ be in $A'\cap {{{\mathcal {L}}}}(v)$ and $A''\cap {{{\mathcal {L}}}}(v)$ respectively, and choose $y',y''$ in $A' \setminus {{{\mathcal {L}}}}(v)$ and $A''\setminus {{{\mathcal {L}}}}(v)$. Note that $x',y'\in R$ because $A'\subseteq R$, and $x''\in R$ because $x''$ is a descendant of $v\in V_1[R]$, and the coloring guarantees that all descendants of nodes in $V_1[R]$ are red.

First, assume both $A'$ and $A''$ are unicolored (that is, both red). Then also $y''\in R$, so $\{x',x'',y',y''\}\subseteq R$ is a compatible set. Note that ${{\,\mathrm{lca}\,}}_1(x',x'') = v \prec {{\,\mathrm{lca}\,}}_1(x',x'',y')$ and similarly ${{\,\mathrm{lca}\,}}_1(x',x'') \prec {{\,\mathrm{lca}\,}}_1(x',x'',y'')$. Since $\{x',x'',y',y''\}$ is compatible, we must also have ${{\,\mathrm{lca}\,}}_2(x',x'') \prec {{\,\mathrm{lca}\,}}_2(x',x'',y')$ and ${{\,\mathrm{lca}\,}}_2(x',x'') \prec {{\,\mathrm{lca}\,}}_2(x',x'',y'')$. But then ${{\,\mathrm{lca}\,}}_2(x',x'')$ is on the path from $x'$ to $y'$ as well as on the path from $x''$ to $y''$. Hence, $A'$ and $A''$ overlap in ${{\,\mathrm{lca}\,}}_2(x',x'')\in V_2$, contradicting that ${\mathcal {P}}'$ does not overlap in $V_2$.

Now, suppose that while $A'$ is unicolored, $A''$ is multicolored . Since $\{x',x'',y'\}\subseteq R$ is compatible, ${{\,\mathrm{lca}\,}}_2(x',x'') \prec {{\,\mathrm{lca}\,}}_2(x',x'',y')$, so the fact that $x',y'\in A'$ implies that ${{\,\mathrm{lca}\,}}_2(x',x'')\in V_2[A']$. Now, it must be the case that ${{\,\mathrm{lca}\,}}_2(A'')\prec {{\,\mathrm{lca}\,}}_2(x',x'')$, because $x''\in A''$ and otherwise $A'$ and $A''$ overlap in ${{\,\mathrm{lca}\,}}_2(x',x'')$, contradicting that ${\mathcal {P}}'$ does not overlap in $V_2$. The fact that $x',x''\in R$ implies that ${{\,\mathrm{lca}\,}}_2(x',x'')\preceq {{\,\mathrm{lca}\,}}_2(R\cup B)$. So ${{\,\mathrm{lca}\,}}_2(A'')\prec {{\,\mathrm{lca}\,}}_2(x',x'')\preceq {{\,\mathrm{lca}\,}}_2(R\cup B)$, and by property (ii), it must thus be the case that ${{\,\mathrm{lca}\,}}_2(x',x'')$ is covered only by components in ${\mathcal {P}}'$ that are subsets of *W* or that are also components of ${\mathcal {P}}$. But this is a contradiction because ${{\,\mathrm{lca}\,}}_2(x',x'')$ is covered by $A'\in {\mathcal {P}}'\setminus {\mathcal {P}}$. $\square$

The next lemma states that the partition resulting after Split is $(R\cup B)$-feasible.

#### Lemma 6

Let ${\mathcal {P}}'$ be the partition after Split$({\mathcal {P}}, (R, B,W))$. Then ${\mathcal {P}}'$ is a refinement of ${\mathcal {P}}$ that is $(R\cup B)$-feasible.

#### Proof

It is easy to see that every component is $(R \cup B\cup \{w\})$-compatible for all $w\in {{{\mathcal {L}}}}$: each component is either unicolored (and thus $(R\cup B\cup \{w\})$-compatible by the fact that the partition is *R*-compatible and *B*-compatible), or it is the result of a Special-Split on a component in which all tricolored triples are compatible, and hence, since all triples in $R\cup B$ are compatible by the fact the component is $(R\cup B)$-compatible , it was already $(R \cup B\cup \{w\})$-compatible for all $w\in {{{\mathcal {L}}}}$ before the Special-Split.

To see that ${\mathcal {P}}'$ does not overlap in $V_2$, note that the fact that ${\mathcal {P}}$ does not overlap in $V_2$ and is splittable (by Lemma 3) implies that $A\cap R, A\cap B, A\cap W$ do not overlap in $V_2$ for any $A\in {\mathcal {P}}$. If *A* is split by a Special-Split into $A\cap R$ and $A\cap (B\cup W)$, then *A* is $(R\cup B\cup \{w\})$-compatible for all $w\in {{{\mathcal {L}}}}$ (again, because *A* has no incompatible tricolored triples and *A* is $(R\cup B)$-compatible ). This implies that there is a node $\hat{u}_r\in V_2$ such that $A\cap {{{\mathcal {L}}}}(\hat{u}_r) = A\cap R$; hence, $A\cap R$ and $A\setminus R$ do not overlap in $V_2$.

It remains to show that no two components in ${\mathcal {P}}'$ overlap in $V_1[R\cup B]$. We check the sufficient conditions in Lemma 5. The only possible multicolored components of ${\mathcal {P}}'$ are bicolored components created by Special-Split on a component in ${\mathcal {P}}$ that is tricolored and in which every tricolored triple is compatible . By property 3 of Lemma 4, the only tricolored components that have a compatible tricolored triple are top components. By Observation 1, the partition at the start of the iteration had at most one tricolored component, and thus there can be at most one top component, say *A*, that is tricolored in ${\mathcal {P}}$. Since *A* is ($R\cup B\cup \{w\}$)-compatible for all $w\in {{{\mathcal {L}}}}$, there is a node $\hat{u}_r\in V_2$ such that $A\cap {{{\mathcal {L}}}}(\hat{u}_r) = A\cap R$. Split subdivides *A* into $A\cap R$ and $A\setminus R$, where $A\setminus R$ is the unique multicolored component in ${\mathcal {P}}'$. Let $A^*=A\setminus R$, and suppose that there exists a component $A'\in {\mathcal {P}}'$ that covers a node $\hat{v}$ on the path from ${{\,\mathrm{lca}\,}}_2(A^*)$ to ${{\,\mathrm{lca}\,}}_2({{{\mathcal {L}}}})$. Then ${{\,\mathrm{lca}\,}}_2(A')$ must be on this path, too, so ${{\,\mathrm{lca}\,}}_2(A^*)\preceq {{\,\mathrm{lca}\,}}_2(A')$. Observe that $A'$ cannot be $A\setminus A^*=A\cap {{{\mathcal {L}}}}(\hat{u}_r)$. Also, since *A* was the unique top component in ${\mathcal {P}}$, no component created in the current iteration has a lowest common ancestor above ${{\,\mathrm{lca}\,}}_2(A)$. So $A'$ must have been a component in the partition at the start of the iteration, and by Lemma 5 we conclude that ${\mathcal {P}}'$ does not overlap in $V_1[R\cup B]$. $\square$

### Find-merge-pair and merge-components

The astute reader may have noted that the Red-Blue Algorithm sometimes increases the number of components by more than necessary to be $(R\cup B)$-feasible. One example of this is given in Fig. 5. More generally, it follows from the arguments in the proof of Lemma 6 that if there is a tricolored component in which every tricolored triple is compatible , then not further subdividing this component would also leave a partition that is $R\cup B$-feasible. Find-Merge-Pair and Merge-Components aim to merge two components of the partition produced at the end of Split, so that the partition with the merged components is still $(R\cup B)$-feasible. Find-Merge-Pair thus looks for a pair of components that can be merged, by scanning the components of the current partition, and finding two leaves in $R\cup B$ that are in different sets of the partition now, but that were in the same component at the start of the current iteration. We note that a pair of components may also be found when no Special-Split is done on a tricolored component in which every tricolored triple is compatible; in other words, Find-Merge-Pair and Merge-Components can do more than simply reversing those splits on tricolored components in which every tricolored triple is compatible . In the proof of the approximation guarantee (in particular, in Proposition 15), we will show the existence of very specific components that can be merged. However, merging any pair of components created in the current iteration leads to the same approximation guarantee.
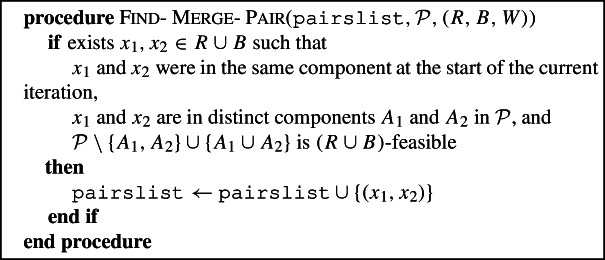


Although we could simply merge the components containing $x_1$ and $x_2$ for the pair found by Find-Merge-Pair, we will not do so until the very end of the algorithm. The reason we keep such “superfluous” splits is because they increase the objective value of the dual solution we use to prove the approximation guarantee of 2 (see Sect. 4). We “reverse” these superfluous splits (i.e., we will merge components) at the end of the algorithm; this is reminiscent of a “reverse delete” in approximation algorithms for network design [13]. The reason to delay these merges is thus to simplify the description of the dual solution in the analysis only.
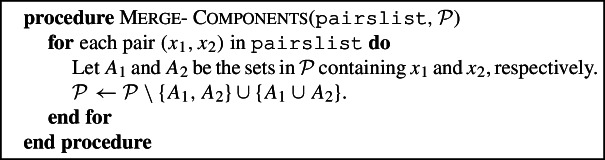


The proof that we will be able to merge the components containing the pair of leaves identified by Find-Merge-Pair at the end of the algorithm will rely on the fact that (i) because the partition is ($R\cup B\cup \{w\}$)-compatible for any $w\in {{{\mathcal {L}}}}$, merging the components containing the identified leaves $x_1,x_2\in R\cup B$ cannot increase the number of incompatible triples contained in a component, and (ii) because the partition is $(R\cup B)$-feasible, future iterations of the algorithm will not further refine the partition induced on $R\cup B$. This is the reason why we do not allow Find-Merge-Pair to choose leaves in *W* (and only choosing leaves in $R\cup B$ is sufficient to prove the claimed approximation guarantee).

#### Lemma 7

Let (*R*, *B*, *W*) be the coloring during some iteration of the Red-Blue Algorithm, and let ${\mathcal {P}}$ be the partition at the end of the iteration. Then the algorithm does not refine the partitioning restricted to $R\cup B$ in later iterations: for any $x,x'\in R\cup B$ that are in the same component of ${\mathcal {P}}$, *x* and $x'$ are in the same component in any partition at any later point of the algorithm’s execution.

#### Proof

Suppose for a contradiction that a later iteration with coloring $(R',B',W')$ separates two leaves $x, x' \in R \cup B$ in the same component of ${\mathcal {P}}$. Let *A* be the component containing *x* and $x'$ at the start of this iteration. Since ${\mathcal {P}}$ is $(R\cup B)$-feasible, no $v\in V_1[R\cup B]$ is a root of infeasibility, and hence all leaves in $R\cup B$, and in particular *x* and $x'$, must have the same color in the coloring $(R',B',W')$. Notice that by the definition of Split, *x* and $x'$ cannot be separated during Split. Hence, they must be separated during Make-$(R\cup B)$-compatible or Make-Splittable. In both cases there must exist some $\hat{u}\in V_2$ such that $A\cap {{{\mathcal {L}}}}(\hat{u})$ is multicolored with respect to the coloring $(R',B',W')$, and $A \cap {{{\mathcal {L}}}}(\hat{u})$ contains precisely one of $x,x'$. By relabeling if needed, assume that $x \in A \cap {{{\mathcal {L}}}}(\hat{u})$ and $x' \in A \setminus {{{\mathcal {L}}}}(\hat{u})$. Let $w \in A \cap {{{\mathcal {L}}}}(\hat{u})$ be any leaf with a color (in the coloring $(R',B',W')$) different from *x*, and note that

1 $$\begin{aligned} {{\,\mathrm{lca}\,}}_2(x,w) \preceq \hat{u} \prec {{\,\mathrm{lca}\,}}_2(x,x',w). \end{aligned}$$

Because all leaves in $R\cup B$, have the same color in $(R',B',W')$, and because *w* has a different color than *x* in $(R',B',W')$, we know that ${{\,\mathrm{lca}\,}}_1(x,x')\prec {{\,\mathrm{lca}\,}}_1(x,x',w)$. But, since ${\mathcal {P}}$ is ($R\cup B\cup \{w\}$)-compatible, this implies that if *w* is in the same component as *x* and $x'$ in (a refinement of) ${\mathcal {P}}$, then ${{\,\mathrm{lca}\,}}_2(x,x')\prec {{\,\mathrm{lca}\,}}_2(x,x',w)$, contradicting (1), because only one of ${{\,\mathrm{lca}\,}}_2(x,x')$ and ${{\,\mathrm{lca}\,}}_2(x,w)$ can be strictly below ${{\,\mathrm{lca}\,}}_2(x,x',w)$.

$$\square $$

### Correctness of the algorithm

#### Theorem 8

The Red-Blue Algorithm returns a feasible solution to MAF.

#### Proof

In each iteration through the main loop of the algorithm, the partition is strictly refined. Thus there are less than $|{{{\mathcal {L}}}}|$ iterations. When the main loop terminates, ${{\,\mathrm{lca}\,}}_1({{{\mathcal {L}}}})$ is not a root of infeasibility , and so the partition at this stage is feasible. It remains to prove that merging components using Merge-Components maintains the feasibility of the partition.

We prove this by induction on *k*, the number of pairs in pairslist. If $k=0$, Merge-Components does nothing, and so the returned partition is indeed feasible.

So suppose $k >0$. Observe that the result of Merge-Components applied to a partition ${\mathcal {P}}$ is the unique finest coarsening of ${\mathcal {P}}$ in which every pair of nodes in pairslist is in the same component, and hence does not depend on the order in which the pairs in pairslist are considered. We may thus assume without loss of generality that they are considered in the reverse order in which they were added to pairslist.

Let ${\mathcal {P}}'$ be the partition obtained during Merge-Components after the components have been merged for all pairs on pairslist, except the pair $(x_1,x_2)$ that was added to pairslist first. Let ${\mathcal {P}}$ be the partition at the moment when $(x_1,x_2)$ was added to pairslist during the main loop of the algorithm, i.e. the partition at the end of Split in the iteration where $(x_1,x_2)$ was added to pairslist; let *R*, *B*, *W* be the three color sets of that iteration. In all subsequent iterations ${\mathcal {P}}$ was further refined, and any of the pairs aside from $(x_1,x_2)$ added to pairslist consists of two leaves that were in the same component in the partition at the start of the iteration in which were they added to pairslist , and hence in the same component of ${\mathcal {P}}$. Thus, ${\mathcal {P}}'$ is a refinement of ${\mathcal {P}}$ and ${\mathcal {P}}'$ is a coarsening of the partition at the end of the last iteration. Thus by Lemma 7, ${\mathcal {P}}$ and ${\mathcal {P}}'$ induce the same partition of $R\cup B$. Moreover, by the induction hypothesis, every component of ${\mathcal {P}}'$ is compatible.

Let $A_1, A_2$ be the components in ${\mathcal {P}}$ containing $x_1,x_2$ respectively. By the choice of $x_1,x_2$, $(A_1\cup A_2)$ is $R\cup B\cup \{w\}$-compatible for every $w\in {{{\mathcal {L}}}}$, and $A_1\cup A_2$ does not overlap any component of ${\mathcal {P}}\setminus \{A_1,A_2\}$ in $V_2\cup V_1[R\cup B]$.

If $A_1, A_2$ are unicolored , they both contain leaves in $R\cup B$ only, because $x_1,x_2\in R\cup B$ by definition of Find-Merge-Pair. As argued above, ${\mathcal {P}}'$ contains components $A_1$ and $A_2$ as well. Furthermore, in this case, the set $A_1\cup A_2$ is a subset of $R\cup B$ and thus $R\cup B\cup \{w\}$-compatibility for all $w\in {{{\mathcal {L}}}}$ implies the set is compatible . Since $V_1[A_1\cup A_2]\subseteq V_1[R\cup B]$, $A_1\cup A_2$ cannot overlap any set $A\in {\mathcal {P}}\setminus \{A_1,A_2\}$; this implies it also does not overlap any set $A'\in {\mathcal {P}}'\setminus \{A_1,A_2\}$, since ${\mathcal {P}}'$ is a refinement of ${\mathcal {P}}$.

If $A_1$ and $A_2$ are not both unicolored , observe that only one of $A_1, A_2$ is bicolored and contains leaves in $B\cup W$, because ${\mathcal {P}}$ does not overlap in $V_1[R\cup B]$ so it can only have one multicolored component, and the only type of multicolored components after Split, are subsets of $B\cup W$. Suppose without loss of generality that $A_1$ is unicolored and $A_2$ contains leaves in $B\cup W$. As mentioned before, by Lemma 7, ${\mathcal {P}}'$ and ${\mathcal {P}}$ have the same components restricted to $R\cup B$, whence ${\mathcal {P}}'$ contains component $A_1$ and a component $A_2'\subseteq A_2$, where $A_2'\cap (R\cup B) = A_2\cap (R\cup B)$.

We need to show that $A_1\cup A_2'$ is compatible and does not overlap any component in ${\mathcal {P}}'\setminus \{A_1,A_2'\}$. For the latter, suppose in order to derive a contradiction that $A_1\cup A_2'$ overlaps $A'\in {\mathcal {P}}'\setminus \{A_1,A_2'\}$. Observe that the only nodes in $V[A_1\cup A_2']$ that are not in $V[A_1]\cup V[A_2']$ are in $V_2\cup V_1[R\cup B]$, so the overlap must be on a node $v\in V_2\cup V_1[R\cup B]$. Since ${\mathcal {P}}'$ is a refinement of ${\mathcal {P}}$, there must exist $A\in {\mathcal {P}}$ such that $A'\subset A$, and thus $A_1\cup A_2'$ overlaps *A* in *v* as well. But then $A_1\cup A_2$ also overlaps *A* in *v* contradicting that ${\mathcal {P}}\setminus \{A_1,A_2\}\cup \{A_1\cup A_2\}$ is $(R\cup B)$-feasible.

To show that $A_1\cup A_2'$ is compatible , note that $A_2'$ is a component of ${\mathcal {P}}'$, and thus, by the induction hypothesis, $A_2'$ is compatible . By the choice of $A_1,A_1$, we know $A_1\cup A_2'\subset A_1\cup A_2$ is ($R\cup B\cup \{w\}$)-compatible for all $w\in {{{\mathcal {L}}}}$. So to show that $A_1\cup A_2'$ is compatible , it suffices to consider $x,w,w'\in A_1\cup A_2'$ with $x\in A_1$ and $w,w'\in A_2'\cap W$. Fix any $x_B{} \in A_2'\cap B$, and note that $x\in R\cup B$. Therefore, ${{\,\mathrm{lca}\,}}_i( x_B{}, w ) = {{\,\mathrm{lca}\,}}_i( x,x_B{},w ) ={{\,\mathrm{lca}\,}}_i( x, w )$ for $i=1,2$, since ${{\,\mathrm{lca}\,}}_i( x, x_B{} ) \prec {{\,\mathrm{lca}\,}}_i( x, x_B{}, w )$ is implied by $A_1\cup A_2$ being $R\cup B\cup \{w\}$-compatible. So $\{x,w,w'\}$ is compatible exactly when $\{x_B{},w,w'\}$ is compatible . Because, as we noted, $A_2'$ is compatible , we conclude that $A_1\cup A_2'$ is compatible . $\square$

## Proof of the approximation guarantee

We showed in the previous section that the Red-Blue Algorithm returns a feasible solution ${\mathcal {P}}$. In order to prove that our algorithm achieves an approximation guarantee of 2, we will use linear programming duality.

### The linear programming relaxation

Let ${{{\mathcal {C}}}}$ be the set of all compatible subsets of ${{{\mathcal {L}}}}$. Introduce a variable $x_L$ for every compatible set $L\in {{{\mathcal {C}}}}$, where in an integral solution, $x_L = 1$ indicates that *L* forms part of the solution to MAF. The constraints ensure that in an integral solution, $\{ L : x_L = 1\}$ is a partition, and that $V[L] \cap V[L'] = \emptyset$ for two distinct sets $L, L'$ with $x_L = x_{L'} = 1$. The objective encodes the size of the partition minus 1. 
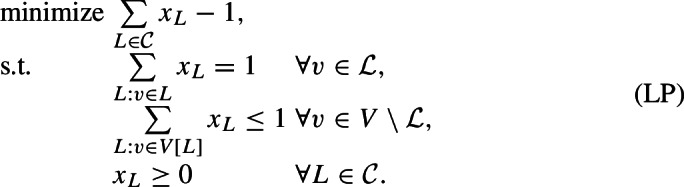


The equality constraint on the leaves can be replaced by the inequalities $\sum _{L:v \in L} x_L \ge 1$ for all $v \in {{{\mathcal {L}}}}$. For given a solution ${\tilde{x}}$ for which the constraint for some leaf *v* is not tight, we can simply choose some set *L* containing *v* with ${\tilde{x}}_L > 0$, and decrease ${\tilde{x}}_L$ while (if $|L| > 1$) increasing ${\tilde{x}}_{L \setminus \{v\} }$. This cannot increase the cost of the solution, and clearly maintains feasibility. By repeating this process, we obtain a solution to (LP) of cost no larger than the cost of the original ${\tilde{x}}$.

In fact, it will be convenient for our analysis to expand the first set of constraints (in their inequality rather than equality form) to contain a constraint for every (not necessarily compatible) set of leaves *A*, stating that every such set must be intersected by at least one component in the chosen MAF solution. All these constraints of this expanded set are clearly implied by the constraints for *A* a singleton, which are exactly the first set of constraints in (LP). 
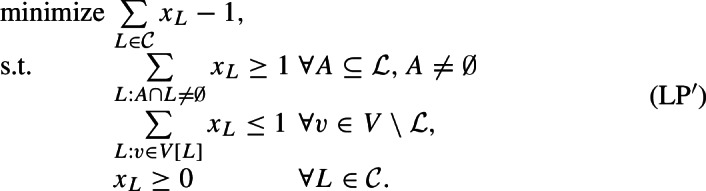
 This expanded formulation provides us a more expressive dual: 
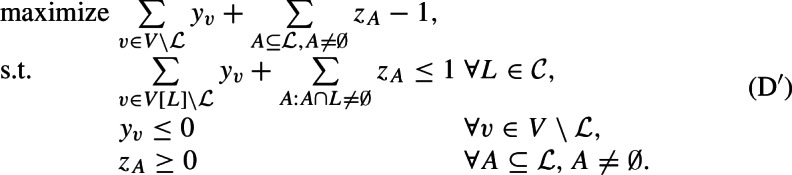
 We will refer to the left-hand side of the first family of constraints, i.e., $\sum _{v\in V[L] \setminus {{{\mathcal {L}}}}} y_v +\sum _{A:A\cap L\ne \emptyset }z_A$, as the *load* on set *L*, and denote it by ${{\,\mathrm{load}\,}}_{(y,z)}(L)$. By weak duality, we have that the objective value of any feasible dual solution provides a lower bound on the objective value of any feasible solution to (LP), and hence also on the optimal value of any feasible solution to MAF. Hence, in order to prove that an agreement forest that has $|{\mathcal {P}}|$ components is a 2-approximation, it suffices to find a feasible dual solution with objective value $\frac{1}{2}(|{\mathcal {P}}|-1)$, i.e., for every new component created by the algorithm, the dual objective value should increase by $\frac{1}{2}$ (on average).

### The dual solution

The dual solution maintained is as follows. Throughout the main loop of the algorithm, $z_A=1$ if and only if *A* is a component in ${\mathcal {P}}$. In the last part of the algorithm, when we merge components according to pairslist , we do not update the dual solution; these operations affect the primal solution (i.e., ${\mathcal {P}}$) only.

Initially, $$y_v = 0 $$ for all $$v\in (V_1\cup V_2)\setminus {{{\mathcal {L}}}}$$. At the start of each iteration, we decrease $$y_{u}$$ by 1, where $$u={{\,\mathrm{lca}\,}}_1(R\cup B)$$. Whenever in the algorithm we choose a component *A* and a node $$\hat{u}\in V_2[A]$$, and separate the component *A* into $$A\cap {{{\mathcal {L}}}}(\hat{u})$$ and $$A\setminus {{{\mathcal {L}}}}(\hat{u})$$, we decrease $$y_{\hat{u}}$$ by 1. To be precise this happens in Make-$$(R\cup B)$$ -compatible, Make-splittable and in one case in Special-Split (where we actually further refine $$A\cup {{{\mathcal {L}}}}(\hat{u})$$). The lines where such nodes are chosen are indicated by $$\star $$ in the description of the algorithm and the procedures it contains.

#### Lemma 9

The dual solution maintained by the algorithm is feasible.

#### Proof

We prove the lemma by induction on the number of iterations. Initially, $z_A=0$ for all $A\ne {{{\mathcal {L}}}}$ and $z_{{{\mathcal {L}}}}=1$ and hence every compatible set *L* has a load of 1.

At the start of an iteration, we decrease $y_{{{\,\mathrm{lca}\,}}_1(R\cup B)}$ by 1, thus decreasing the load by 1 on any multicolored compatible set *L*. We show that the remainder of the iteration increases the load by at most 1 on a multicolored compatible set and that it does not increase the load on any unicolored compatible set.

First, observe that Make-$(R\cup B)$ -compatible and Make-Splittable do not increase the load on any set: Separating *A* into $A\cap {{{\mathcal {L}}}}(\hat{u})$ and $A\setminus {{{\mathcal {L}}}}(\hat{u})$ increases the load on sets *L* that intersect both $A\cap {{{\mathcal {L}}}}(\hat{u})$ and $A\setminus {{{\mathcal {L}}}}(\hat{u})$, since $z_A$ gets decreased from 1 to 0, and $z_{A\cap {{{\mathcal {L}}}}(\hat{u})}$ and $z_{A\setminus {{{\mathcal {L}}}}(\hat{u})}$ increase from 0 to 1. However, in this case $\hat{u}\in V[L]$, and thus decreasing $y_{\hat{u}}$ by 1 ensures that the load on *L* does not increase.

To analyze the effect of Split, we use the following two claims.

#### Claim 10

In the procedure Split$({\mathcal {P}}, (R, B, W))$ the load on any compatible set *L* is increased by at most the number of components $A\in {\mathcal {P}}$ such that $L\cap A$ is multicolored .

*Proof.* If the load on *L* is increased because Split splits a bicolored component *A* into two unicolored components, then *L* must intersect both new components, so $L\cap A$ is bicolored (and thus multicolored ) and the load on *L* is increased by 1.

Consider the case where the load on *L* is increased because a tricolored component *A* is split into $A\cap R$, $A\cap B$ and $A\cap W$. This split happens when *all* tricolored triples in *A* are incompatible . Therefore $L\cap A$ cannot be tricolored . Since the load on *L* increased by splitting *A*, we conclude that $L\cap A$ must be bicolored and the load on *L* is increased by 1.

Finally, suppose the load on *L* is increased because Special-Split($A, {\mathcal {P}}, (R, B, W)$) is executed for a component *A*. We consider the two cases of Special-Split. In the first case, *A* is split into two components, one of which contains all red leaves in *A*. The load on a set *L* thus increases by 1 if $L\cap A$ is multicolored and $L\cap A\cap R\ne \emptyset$ and by 0 otherwise. In the second case, *A* is split into four components; we think of this as first splitting *A* into $A\cap {{{\mathcal {L}}}}(\hat{u})$ and $A\setminus {{{\mathcal {L}}}}(\hat{u})$, and then splitting $A\cap {{{\mathcal {L}}}}(\hat{u})$ by intersecting with *R*, *B* and *W*. Since $y_{\hat{u}}$ is decreased by 1, splitting *A* into $A\cap {{{\mathcal {L}}}}(\hat{u})$ and $A\setminus {{{\mathcal {L}}}}(\hat{u})$ does not affect the load on any set *L*. Splitting $A\cap {{{\mathcal {L}}}}(\hat{u})$ by intersecting with *R*, *B*, *W* increases the load on *L* by 1 if $L\cap A\cap {{{\mathcal {L}}}}(\hat{u})$ is bicolored and by 2 if it is tricolored . We show below that $L\cap A\cap {{{\mathcal {L}}}}(\hat{u})$ cannot be tricolored , which implies that the load on *L* increases by at most 1 if $A\cap L$ is multicolored , thus proving the claim. Suppose $L\cap A\cap {{{\mathcal {L}}}}(\hat{u})$ contains a triple $x_B\in B, x_R\in R, x_W\in W$. The fact that *A* is $(R\cup B)$-compatible implies that ${{\,\mathrm{lca}\,}}_2(x_B, x_R) = {{\,\mathrm{lca}\,}}_2(A\cap (R\cup B))=\hat{u}$. Since $x_W\in {{{\mathcal {L}}}}(\hat{u})$, we thus have either ${{\,\mathrm{lca}\,}}_2(x_B, x_W)\prec \hat{u}={{\,\mathrm{lca}\,}}_2(x_B, x_R)$ or ${{\,\mathrm{lca}\,}}_2(x_R, x_W)\prec \hat{u}={{\,\mathrm{lca}\,}}_2(x_B, x_R)$. In either case, $\{x_B, x_R, x_W\}$ is incompatible, contradicting that *L* is compatible. $\diamond$

#### Claim 11

If *L* is compatible, and *A* and $A'$ do not overlap in $V_2$, then $L\cap A$ and $L\cap A'$ cannot both be multicolored .

*Proof.* Assume that $|A|\ge 2, |A'|\ge 2$ (otherwise, the claim is vacuously true). Since $V_2[A]$ and $V_2[A']$ are disjoint, we may assume without loss of generality that ${{\,\mathrm{lca}\,}}_2(x,y)\prec {{\,\mathrm{lca}\,}}_2(x,y,x')$ for all $x,y\in A$ and $x'\in A'$. Hence, if $L\cap A$ and $L\cap A'$ are both multicolored sets, then there exist $x,y,x',y'\in L$ where *x*, *y* have different colors, $x',y'$ have different colors, ${{\,\mathrm{lca}\,}}_2(x,y)\prec {{\,\mathrm{lca}\,}}_2(x,y,x')$, and ${{\,\mathrm{lca}\,}}_2(x,y)\prec {{\,\mathrm{lca}\,}}_2(x,y,y')$. We claim this implies $\{x,y,x',y'\}$ is incompatible, a contradiction since $x,y,x',y'\in L$ and *L* is compatible.

Clearly one of *x*, *y* has the same color as one of $x',y'$. Suppose without loss of generality that $x,x'$ have the same color. If *x* and $x'$ are both red, *y* is either blue or white. *x* and $x'$ being red implies ${{\,\mathrm{lca}\,}}_1(x,x')\prec {{\,\mathrm{lca}\,}}_1(x,y,x')$, which, since ${{\,\mathrm{lca}\,}}_2(x,y)\prec {{\,\mathrm{lca}\,}}_2(x,y,x')$, shows that $\{x,x',y\}$ is an incompatible triple. The case when *x* and $x'$ are blue is analogous. If *x* and $x'$ are both white, then *y* and $y'$ are in $R\cup B$. This implies ${{\,\mathrm{lca}\,}}_1(y,y')\prec {{\,\mathrm{lca}\,}}_1(x,y,y')$, and so, since ${{\,\mathrm{lca}\,}}_2(x,y)\prec {{\,\mathrm{lca}\,}}_2(x,y,y')$, this implies $\{x,y,y'\}$ is an incompatible triple. $\diamond$

It follows immediately from the two claims that Split increases the load by at most 1 on any multicolored compatible set and that it does not increase the load on any unicolored set, which completes the proof of the lemma. $\square$

### The primal and dual objective values

Let ${\mathcal {P}}$, pairslist be the partition and pairslist at the end of an iteration, and let $D=\sum _{v\in V\setminus {{{\mathcal {L}}}}} y_v + |{\mathcal {P}}|-1$ be the objective value of the dual solution at this time. In this section, we show that every iteration of our algorithm maintains the invariant that

2 $$\begin{aligned} 2D\ge \left( |{\mathcal {P}}|-1-|\mathtt{pairslist} |\right) . \end{aligned}$$

Observe that the approximation guarantee immediately follows from this inequality, since the objective value of the algorithm’s solution is ${\mathcal {P}}-1-|\mathtt{pairslist} |$ (where ${\mathcal {P}}$, pairslist are the partition and pairslist at the end of the final iteration), and by weak duality *D* gives a lower bound on the optimal value of the MAF instance.

To prove that the algorithm maintains the invariant, we will show that a given iteration increases the left-hand side of (2) by at least as much as the right-hand side. We let $\Delta D$ be the change in the dual objective during the iteration and $\Delta P$ be the increase in the number of components minus the number of pairs added to pairslist (either 0 or 1) during the current iteration.

Since at the start of the algorithm, the partition consists of exactly one component, and $y_v=0$ for all $v\in V\setminus {{{\mathcal {L}}}}$, (2) holds before the first iteration. So to show (2), it suffices to show that

3 $$\begin{aligned} 2\Delta D \ge \Delta P \end{aligned}$$

for any iteration.

In what follows, we use the following to refer to the state of the partition at various points in the current iteration: ${\mathcal {P}}^{(0)}$ at the start; ${\mathcal {P}}^{(1)}$ after Make-$(R\cup B)$ -compatible; ${\mathcal {P}}^{(2)}$ after Make-Splittable; and ${\mathcal {P}}^{(3)}$ after Split.

We begin by showing that the coloring (*R*, *B*, *W*) and the partition ${\mathcal {P}}^{(0)}$ satisfy the conditions of one of three cases.

#### Lemma 12

Given an infeasible partition ${\mathcal {P}}^{(0)}$ that does not overlap in $V_2$, let $u\in V_1$ be a lowest root of infeasibility, and let $u_\ell$ and $u_r$ be *u*’s children in $T_1$. Let $R={{{\mathcal {L}}}}(u_r), B={{{\mathcal {L}}}}(u_\ell )$, and $W={{{\mathcal {L}}}}\setminus (R\cup B)$. Then ${\mathcal {P}}^{(0)}$ is *R*-compatible and *B*-compatible and satisfies exactly one of the following three additional properties:

*Case 1*: ${\mathcal {P}}^{(0)}$ has exactly one multicolored component, say $A_0$, where $A_0$ is tricolored , not $(R\cup B)$-compatible , and there exists $x_W\in A_0\setminus {{{\mathcal {L}}}}({{\,\mathrm{lca}\,}}_2(A_0\cap (R\cup B)))$, i.e., $A_0$ contains a compatible tricolored triple.
*Case 2*: ${\mathcal {P}}^{(0)}$ has exactly two multicolored components, say $A_B, A_R$, where $A_B\cap R=\emptyset$ and $A_R\cap B=\emptyset$.
*Case 3*: ${\mathcal {P}}^{(0)}$ has exactly one multicolored component, say $A_0$, where $A_0$ is tricolored , $(R\cup B)$-compatible and $A_0$ contains no compatible tricolored triple.

We will see in the proof below that Cases 1, 2 and 3 correspond to a lowest root of infeasibility satisfying (a), (b) and (c) respectively in Definition 4. We refer the reader to Fig. 1 for an illustration of the three cases.

#### Proof

Observe that if ${\mathcal {P}}^{(0)}$ is infeasible, then the root of $T_1$, i.e., ${{\,\mathrm{lca}\,}}_1({{{\mathcal {L}}}})$ is a root of infeasibility, and that no $v\in {{{\mathcal {L}}}}$ is a root of infeasibility. Hence, *u* is well-defined and *R* and *B* are non-empty. Note that ${\mathcal {P}}^{(0)}$ is *R*-compatible and *B*-compatible, since *u*’s children are not roots of infeasibility.

We will show that if *u* satisfies condition (a) in the definition of a root of infeasibility, then the conditions of Case 1 are satisfied, if (b) holds, the conditions of Case 2 are satisfied, and if (c) holds, then the conditions of Case 3 are satisfied.

We start with (b): ${\mathcal {P}}^{(0)}$ overlaps in $V_1[{{{\mathcal {L}}}}(u)]$. Observe that, because *u* is a lowest root of infeasibility, the only node in $V_1[{{{\mathcal {L}}}}(u)]$ on which ${\mathcal {P}}^{(0)}$ overlaps is *u*, and thus there must be at least two multicolored components if (b) holds. If there are two multicolored components, both containing, say, red leaves, then they overlap in $u_r={{\,\mathrm{lca}\,}}_1(R)$, which implies $u_r$ is a root of infeasibility, contradicting the choice of *u*. Similarly, there is at most one multicolored component containing blue leaves. Hence, the conditions of Case 2 are satisfied.

If (b) does not hold, i.e., the partition does not overlap in $V_1[{{{\mathcal {L}}}}(u)]$, then there is at most one multicolored component; the conditions in (a) and (c) both imply there is at least one. Thus there is exactly one multicolored component, which we will call $A_0$. We let $R_0=R\cap A_0, B_0=B\cap A_0$ and $\hat{u}={{\,\mathrm{lca}\,}}_2(R_0\cup B_0)={{\,\mathrm{lca}\,}}_2(A_0\cap (R\cup B))$ (where we stress that $\hat{u}$ is a node in $V_2$, whereas *u* is a node in $V_1$).

If (a) holds, then $A_0$ is not $(R\cup B)$-compatible , and thus $R_0\ne \emptyset , B_0\ne \emptyset$. To derive a contradiction, suppose that Case 1 is not implied, i.e., there does not exist $x_W\in A_0\setminus {{{\mathcal {L}}}}({{\,\mathrm{lca}\,}}_2(A_0\cap (R\cup B)))$, i.e., $A_0\subseteq {{{\mathcal {L}}}}(\hat{u})$. Observe that, because $A_0$ is not $(R\cup B)$-compatible , ${{\,\mathrm{lca}\,}}_2(R_0)=\hat{u}$ or ${{\,\mathrm{lca}\,}}_2(B_0) = \hat{u}$. Suppose the former holds without loss of generality. But then ${{\,\mathrm{lca}\,}}_1(R_0)$ is a root of infeasibility satisfying (c), because $A_0\setminus R_0\ne \emptyset$, and for all $w\in A_0\setminus R_0$, $R_0\cup \{w\}$ is incompatible, by the fact that $w\in {{{\mathcal {L}}}}({{\,\mathrm{lca}\,}}_2(R_0))$, and $w\not \in {{{\mathcal {L}}}}({{\,\mathrm{lca}\,}}_1(R_0))$. But ${{\,\mathrm{lca}\,}}_1(R_0)$ is a descendant of *u*, thus contradicting the choice of *u*.

Suppose now (c) holds, i.e., ${\mathcal {P}}^{(0)}$ is $(R\cup B)$-compatible , and in particular $A_0$ is $(R\cup B)$-compatible . Because $A_0$ is multicolored , we can assume without loss of generality that $R_0\ne \emptyset$. If $B_0=\emptyset$, then (c) holds for ${{\,\mathrm{lca}\,}}_1(R_0)$, which is a descendant of *u*, thus contradicting the choice of *u*. Since $A_0\setminus (R_0\cup B_0)\ne \emptyset$ by condition (c), we conclude $A_0$ is tricolored . It remains to show every tricolored triple is incompatible. Suppose for a contradiction that $\{x, y, w\}\in A_0$ is a tricolored triple that is compatible. Let *w* be the white leaf in the triple, then compatibility requires that ${{\,\mathrm{lca}\,}}_2(x,y) \prec {{\,\mathrm{lca}\,}}_2(x,y,w)$. On the other hand, the fact that $A_0$ is $(R\cup B)$-compatible implies that ${{\,\mathrm{lca}\,}}_2(x,y) = {{\,\mathrm{lca}\,}}_2(R_0\cup B_0)$. But then any tricolored triple in $A_0$ containing *w* is compatible, so that $A\cap (R\cup B)\cup \{w\}$ is compatible, contradicting that condition (c) holds. $\square$

Recall that the coloring is defined only at the start of the iteration. The lemma ensures that the partitions during the iteration always have either one (in Cases 1 and 3) or two (in Case 2) top components. Furthermore, we can use the lemma to show that the components created by Make-$(R\cup B)$ -compatible and Make-Splittable are multicolored .

#### Lemma 13

Only multicolored components are created by Make-$(R\cup B)$ -compatible and Make-Splittable, i.e., if $A\in {\mathcal {P}}^{(2)}\setminus {\mathcal {P}}^{(0)}$, then *A* is multicolored.

#### Proof

It follows immediately from the description of Make-Splittable that components created by this procedure are multicolored . Observe that Make-$(R\cup B)$ -compatible is used only if ${\mathcal {P}}^{(0)}$ is not $(R\cup B)$-compatible. By Lemma 12, this implies ${\mathcal {P}}^{(0)}$ must have exactly one multicolored component $A_0$, which contains a white leaf $x_W$ that is not a descendent of ${{\,\mathrm{lca}\,}}_2(A_0)\cap (R\cup B))$. From the description of Make-$(R\cup B)$ -compatible, we (possibly repeatedly) subdivide the top component $A_0$ into $A_0\cap {{{\mathcal {L}}}}(\hat{u})$ and $A_0\setminus {{{\mathcal {L}}}}(\hat{u})$. From the description of Make-$(R\cup B)$ -compatible, it is clear that the newly created non-top component $A_0\cap {{{\mathcal {L}}}}(\hat{u})$ intersects both *R* and *B*, and the new top component $A_0\setminus {{{\mathcal {L}}}}(\hat{u})$ must have a leaf in $R\cup B$, because otherwise $A_0$ is already $(R\cup B)$-compatible. So $\hat{u}\preceq {{\,\mathrm{lca}\,}}_2(A_0\cap (R\cup B))$, and $x_W$ must also be in the new top component $A_0\setminus {{{\mathcal {L}}}}(\hat{u})$, thus ensuring that the top component remains multicolored . $\square$

For Cases 2 and 3, the analysis is quite simple.

#### Proposition 14

Let the initial partition ${\mathcal {P}}^{(0)}$ and coloring (*R*, *B*, *W*) satisfy the conditions of Cases 2 or 3 in Lemma 12. Then $2\Delta D \ge \Delta P$.

#### Proof

We first make two observations that apply in Cases 2 and 3: (i) ${\mathcal {P}}^{(0)}$ is already $(R\cup B)$-compatible , so ${\mathcal {P}}^{(1)}={\mathcal {P}}^{(0)}$, and (ii) Split(${\mathcal {P}}^{(2)},(R,B,W)$) will not perform any Special-Split, because no component of a refinement of ${\mathcal {P}}^{(0)}$ can have a tricolored triple that is compatible (since we are in Case 2 or 3). From these two observations we derive that

4 $$\begin{aligned} |{\mathcal {P}}^{(3)}| - |{\mathcal {P}}^{(2)}| = |{\mathcal {P}}^{(2)}|-|{\mathcal {P}}^{(0)}|+2. \end{aligned}$$

To see this, note that, since no Special-Split is performed, $|{\mathcal {P}}^{(3)}|-|{\mathcal {P}}^{(2)}|$ is equal to the number of bicolored components in ${\mathcal {P}}^{(2)}$ plus twice the number of tricolored components in ${\mathcal {P}}^{(2)}$. By Lemma 13${\mathcal {P}}^{(2)}$ has $|{\mathcal {P}}^{(2)}|-|{\mathcal {P}}^{(0)}|$ more multicolored components than ${\mathcal {P}}^{(0)}$, and, since ${\mathcal {P}}^{(1)}={\mathcal {P}}^{(0)}$, property 2 of Lemma 4 implies that ${\mathcal {P}}^{(2)}$ has the same number of tricolored components as ${\mathcal {P}}^{(0)}$. So in Case 2, ${\mathcal {P}}^{(2)}$ has $|{\mathcal {P}}^{(2)}|-|{\mathcal {P}}^{(0)}| +2$ bicolored components and zero tricolored components, and in Case 3, ${\mathcal {P}}^{(2)}$ has $|{\mathcal {P}}^{(2)}|-|{\mathcal {P}}^{(0)}|$ bicolored components plus one tricolored component, and indeed (4) holds.

In addition, we note that

5 $$\begin{aligned} \Delta D =|{\mathcal {P}}^{(3)}|-|{\mathcal {P}}^{(2)}|-1. \end{aligned}$$

To see this, note that at the start of the iteration, the dual objective value is reduced by 1 when $y_{u}$ is decreased by 1 for $u={{\,\mathrm{lca}\,}}_1(R\cup B)$. Make-splittable does not change the dual objective value, because, even though $|{\mathcal {P}}|$ increases by 1 every time the number of components increases by 1, $\sum _v y_v$ decreases by 1 as well. Finally, since Split will not perform any Special-Split, the increase in the dual objective value due to Split is equal to the increase in the number of components due to Split, which is $|{\mathcal {P}}^{(3)}|-|{\mathcal {P}}^{(2)}|$.

Note that the size of pairslist may increase but will never decrease, and thus

$$\begin{aligned} \Delta P&\le |{\mathcal {P}}^{(3)}|-|{\mathcal {P}}^{(2)}|+|{\mathcal {P}}^{(2)}|-|{\mathcal {P}}^{(0)}|&\\&= 2\left( |{\mathcal {P}}^{(3)}|-|{\mathcal {P}}^{(2)}|\right) - 2&\text{ by } (4)\\&= 2\Delta D&\text{ by } (5). \end{aligned}$$

$$\square $$

We now prove a similar proposition for Case 1, the proof of which is more involved.

#### Proposition 15

Suppose the initial partition ${\mathcal {P}}^{(0)}$ and coloring (*R*, *B*, *W*) satisfy the conditions of Case 1 in Lemma 12. Then $2\Delta D \ge \Delta P$.

#### Proof

In Case 1, we start with ${\mathcal {P}}^{(0)}$ containing one tricolored component $A_0$, which is not $(R\cup B)$-compatible . $A_0$ is the only component that will be subdivided in the current iteration (by property 1 of Lemma 4). Note that ${\mathcal {P}}^{(1)}$ and ${\mathcal {P}}^{(2)}$ therefore have exactly one top component.

Let $x_W$ be a white leaf in $A_0$ that is not a descendant of ${{\,\mathrm{lca}\,}}_2(A_0\cap (R\cup B))$, which exists by the definition of Case 1. By property 5 in Lemma 4, $x_W$ is contained in the top component of ${\mathcal {P}}^{(2)}$, and by Lemma 13 this component is multicolored . Therefore, the top component of ${\mathcal {P}}^{(2)}$ is either bicolored , or it is tricolored and a Special-Split is performed on the top component.

Let $\chi$ be an indicator variable that is 1 if the top component in ${\mathcal {P}}^{(2)}$ is tricolored and has a tricolored triple that is incompatible ; in other words, $\chi =1$ if Special-Split subdivides the top component into four components. If $\chi =0$, then either the top component is bicolored or it is tricolored and all its triplets are compatible ; in other words, $\chi =0$ if the top component is subdivided into two components by Split (possibly via Special-Split). Thus splitting the top component increases the number of components by $1+2\chi$.

Now, let *t* be the number of tricolored components in ${\mathcal {P}}^{(2)}$ that are not top components. We claim that

6 $$\begin{aligned} |{\mathcal {P}}^{(3)}|-|{\mathcal {P}}^{(2)}| = |{\mathcal {P}}^{(2)}|-|{\mathcal {P}}^{(0)}|+1+2\chi + t. \end{aligned}$$

To show this, we need to argue that the increase in the number of components due to splitting the multicolored non-top components is $|{\mathcal {P}}^{(2)}|-|{\mathcal {P}}^{(0)}|+t$. Since ${\mathcal {P}}^{(0)}$ has one multicolored component, Lemma 13 implies that ${\mathcal {P}}^{(2)}$ has $|{\mathcal {P}}^{(2)}|-|{\mathcal {P}}^{(0)}|+1$ multicolored components. Precisely one of these is a top component, so ${\mathcal {P}}^{(2)}$ has $|{\mathcal {P}}^{(2)}|-|{\mathcal {P}}^{(0)}|$ multicolored non-top components. By property 3, each of the tricolored components that are not top components do not require a Special-Split and are thus subdivided into three components by Split. Hence, splitting the components that are not top components increases the number of components by $|{\mathcal {P}}^{(2)}|-|{\mathcal {P}}^{(0)}|+t$.

Next, we analyze the increase in the dual objective. We claim that

7 $$\begin{aligned} \Delta D = |{\mathcal {P}}^{(3)}|-|{\mathcal {P}}^{(2)}| - 1 -\chi . \end{aligned}$$

To see this, note that the dual objective is decreased by 1 when we decrease $y_{{{\,\mathrm{lca}\,}}_1(R\cup B)}$ by 1 at the start of the iteration. As argued in the proof of the previous proposition, the dual objective is not affected by Make-Splittable. The same argument used there implies that the same holds for Make-$(R\cup B)$ -compatible. Finally, if $\chi = 0$, the increase in the dual objective due to Split is equal to the increase in the number of components, $|{\mathcal {P}}^{(3)}|-|{\mathcal {P}}^{(2)}|$. If $\chi =1$, the same holds, but Special-Split on the top component also decreases $y_{\hat{u}_0}$ by 1.

So we get that

$$\begin{aligned} |{\mathcal {P}}^{(3)}|-|{\mathcal {P}}^{(0)}|&= |{\mathcal {P}}^{(3)}|-|{\mathcal {P}}^{(2)}| + |{\mathcal {P}}^{(2)}|-|{\mathcal {P}}^{(0)}|\\&= 2\left( |{\mathcal {P}}^{(3)}|-|{\mathcal {P}}^{(2)}|\right) -1-2\chi - t&\text{ by } (6)\\&= 2\Delta D + 1 - t&\text{ by } (7). \end{aligned}$$

Hence, if $t\ge 1$ we have $\Delta P \le 2\Delta D$ as required. So the rest of the proof, which requires quite some extra technicalities, deals with the situation of Case 1 and $t=0$. Recall that $\Delta P$ is equal to $|{\mathcal {P}}^{(3)}|-|{\mathcal {P}}^{(0)}|$ minus the number of pairs added to pairslist in the current iteration; hence, to conclude that $\Delta P \le 2\Delta D$ if $t=0$, we need to show a pair is added to $\mathtt{pairslist} {}$ by Find-Merge-Pair.

We will say that a component $A\in {\mathcal {P}}^{(3)}$ is able to *reach* $\hat{u}$ if $\hat{u}\in V_2[A]$ or if ${{\,\mathrm{lca}\,}}_2(A)\prec \hat{u}$ and all intermediate nodes on the path from ${{\,\mathrm{lca}\,}}_2(A)$ to $\hat{u}$ are not covered by any component in ${\mathcal {P}}^{(3)}$. The following lemma (which is actually valid in general, and not only for Case 1) enumerates precisely the situations when a merge is possible. $\square$

#### Lemma 16

Let $A_0 \in {\mathcal {P}}^{(0)}$, and let ${\mathcal {Q}}$ denote the set of components in ${\mathcal {P}}^{(3)}$ that are subsets of $A_0$. Then there exists a pair of elements in $A_0$ that can be added to pairslist if and only if at least one of the following is true:

- ${\mathcal {Q}}$ contains a bicolored component.
- There is a node $\hat{u}\in V_2$ that can be reached by two red components or two blue components in ${\mathcal {Q}}$.
- There is a node $\hat{u}\in V_2$ that can be reached by a red and a blue component in ${\mathcal {Q}}$, but is not covered by these components. Furthermore, the node $\hat{u}$ must satisfy that the nodes on the path from $\hat{u}$ to ${{\,\mathrm{lca}\,}}_2(A_0)$ are not covered by any red or blue component in ${\mathcal {Q}}$.

#### Proof

Since any two multicolored components overlap in ${{\,\mathrm{lca}\,}}_1(R\cup B)$ and ${\mathcal {P}}^{(2)}$ does not overlap in ${{\,\mathrm{lca}\,}}_1(R\cup B)$ by Lemma 6, there is at most one tricolored component in ${\mathcal {P}}^{(2)}$. By the definitions of Split and Special-Split, ${\mathcal {P}}^{(3)}$ therefore has at most one multicolored component, which has blue and white leaves and is created by applying Special-Split to the tricolored component in ${\mathcal {P}}^{(2)}$. If this blue-white component exists in ${\mathcal {Q}}$, we denote it by $A^*$.

- If ${\mathcal {Q}}$ contains a bicolored component $A^*$, let $A\cup A^*$ be the tricolored component from which Special-Split formed a red component *A* and the bicolored component $A^*$. We show that we can merge *A* and $A^*$, which boils down to undoing the Special-Split operation, to obtain a new partition that is $(R\cup B)$-feasible. Since $A\cup A^*$ was not overlapping with any other component in $V_2$, undoing the Special-Split yields a component that does not overlap any other component of the partition in $V_2$. For every $w\in W$, $A\cup A^*$ is ($R\cup B\cup \{w\}$)-compatible since $A\cup A^*$ is $(R\cup B)$-compatible and, by the conditions of the Special-Split operation, every tricolored triple in $A\cup A^*$ is compatible. Since $A\cup A^*$ was the unique top component in ${\mathcal {P}}^{(2)}$, any component of ${\mathcal {P}}^{(2)}$ (and hence of its refinement ${\mathcal {P}}^{(3)}$) overlapping a node $\hat{v}$ such that ${{\,\mathrm{lca}\,}}_2(A\cup A^*)\prec \hat{v}\preceq {{\,\mathrm{lca}\,}}_2(R\cup B)$ must be a component in ${\mathcal {P}}^{(0)}$. Therefore, by Lemma 5, the new partition does not overlap in $V_1[R\cup B]$.
- If ${\mathcal {Q}}$ does not contain a bicolored component $A^*$, suppose $A, A' \in {\mathcal {Q}}$ are distinct red components in ${\mathcal {Q}}$ so that *A* and $A'$ can both reach the same node $\hat{u}$ in $V_2$. Then merging *A* and $A'$ gives a new partition that does not overlap in $V_2$, and which has no multicolored components. Since $A_0 \cap R$ is compatible, so is $A \cup A'$. By Lemma 5 and the fact that the new partition does not have any multicolored components, it does not overlap in $V_1[R\cup B]$. Hence, merging *A* and $A'$ gives a new partition that is $(R\cup B)$-feasible.
  The same applies if *A* and $A'$ are both blue components in ${\mathcal {Q}}$.
- If ${\mathcal {Q}}$ does not contain a bicolored component $A^*$, suppose there exist $A, A' \in {\mathcal {Q}}$ with *A* red and $A'$ blue such that (i) there exists $\hat{u}\in V_2 \setminus (V_2[A] \cup V_2[A'])$ that can be reached by both *A* and $A'$; and (ii) the nodes on the path from $\hat{u}$ to ${{\,\mathrm{lca}\,}}_2(A_0)$ are not in $V_2[A'']$ for any red or blue component $A''$ in ${\mathcal {Q}}$. Observe that (ii) implies that any component $A''$ such that $V_2[A'']$ contains nodes on the path from $\hat{u}$ to ${{\,\mathrm{lca}\,}}_2(A_0)$ must be subsets of *W*: $A''$ must be in ${\mathcal {Q}}$ if $V_2[A'']$ contains a node on this path, and by the case assumption, ${\mathcal {Q}}$ contains no multicolored component.
  Merging *A* and $A'$ gives a new partition that does not overlap in $V_2$ and the new component $A\cup A'$ is $(R\cup B)$-compatible by (i). Thus the new partition is $(R\cup B)$-compatible , and since it has no components with white leaves as well as leaves in $R\cup B$, it is vacuously also ($R\cup B\cup \{w\}$)-compatible for any $w\in {{{\mathcal {L}}}}$. $A\cup A'$ is the unique bicolored component in this new partition, thus satisfying condition (i) of Lemma 5. Moreover, it satisfies that any node on the path from $\hat{u}={{\,\mathrm{lca}\,}}_2(A\cup A')$ to ${{\,\mathrm{lca}\,}}_2(A_0)$ is not covered by a component that is not white. By Lemma 12, $A_0$ must have been the unique multicolored component in ${\mathcal {P}}^{(0)}$, and thus the components of the partition that overlap a node on the path from ${{\,\mathrm{lca}\,}}_2(A_0)$ to ${{\,\mathrm{lca}\,}}_2(R\cup B)$ were not changed in the current iteration. Therefore, also condition (ii) of Lemma 5 is satisfied, and the lemma implies that the new partition does not overlap in $V_1[R\cup B]$. Hence, merging *A* and $A'$ gives a new partition that is $(R\cup B)$-feasible.

We note that the above three cases encompass all possible merge opportunities within ${\mathcal {Q}}$. If two components cannot reach the same node $\hat{u}\in V_2$, then merging them gives a partition that overlaps in $V_2$. If red and blue components *A* and $A'$ can only reach nodes in $V_2$ that are covered by either *A* or $A'$, then $A\cup A'$ is not $(R\cup B)$-compatible. And if a red and blue component *A* and $A'$ can reach a node $\hat{u}\in V_2$ that is not in $V_2[A] \cup V_2[A']$, but some node on the path from $\hat{u}$ to ${{\,\mathrm{lca}\,}}_2(A_0)$ is covered by a component $A''\in {\mathcal {Q}}$ that is red or blue, then $A\cup A'$ will overlap $A''$ in $V_1[R]$ or $V_1[B]$. To see this, assume $A''$ is red (the blue case is analogous) and let $\hat{v}$ be the node in $V_2[A'']$ closest to $\hat{u}$ on the path from $\hat{u}$ to ${{\,\mathrm{lca}\,}}_2(A_0)$. Then $\hat{v}={{\,\mathrm{lca}\,}}_2(A\cup (A''\cap {{{\mathcal {L}}}}(\hat{v})) \prec {{\,\mathrm{lca}\,}}_2(A'')$, and since $A\cup A''$ are compatible in *R*, we should also have ${{\,\mathrm{lca}\,}}_1(A\cup (A''\cap {{{\mathcal {L}}}}(\hat{v})) \prec {{\,\mathrm{lca}\,}}_1(A'')$. Thus $A''$ and *A* overlap on a node on the path from ${{\,\mathrm{lca}\,}}_1(A)$ to ${{\,\mathrm{lca}\,}}_1(R\cup B)$. $\square$

We are now ready to complete the proof of Proposition 15, by showing that in Case 1 and if $t=0$ (i.e., if ${\mathcal {P}}^{(2)}$ has no tricolored components that are not top components), then at least one of (a), (b) and (c) in Lemma 16 holds for ${\mathcal {P}}^{(3)}$. By the conditions of Case 1, the unique tricolored component $A_0$ in ${\mathcal {P}}^{(0)}$ is not $(R\cup B)$-compatible, and there exists $x_W\in A_0\setminus {{{\mathcal {L}}}}({{\,\mathrm{lca}\,}}_2(A_0\cap (R\cup B)))$.

If (a) holds, we are done, so suppose (a) does not hold, i.e., ${\mathcal {P}}^{(3)}$ has only unicolored components. We first make some observations which we later use to conclude that (b) or (c) must hold. Let $\hat{u}$ be the last node chosen in Make-$(R\cup B)$ -compatible to subdivide the top component. Because $A_0$ is not $(R\cup B)$-compatible, at least one iteration of Make-$(R\cup B)$ -compatible has to be executed on the component, so the existence of $\hat{u}$ follows. Let $A\subseteq A_0$ be the top component that is subdivided into $A\cap {{{\mathcal {L}}}}(\hat{u})$ and $A\setminus {{{\mathcal {L}}}}(\hat{u})$ at this point (after which the current partition is ${\mathcal {P}}^{(1)}$). We observe some properties of the two new components:

- Letting $\hat{u}_\ell$ and $\hat{u}_r$ be the children of $\hat{u}$, then $A\cap {{{\mathcal {L}}}}(\hat{u}_\ell ) \subseteq B$ and $A\cap {{{\mathcal {L}}}}(\hat{u}_r) \subseteq R$. To see this, note that by definition of Make-$(R\cup B)$ -compatible, $A\cap {{{\mathcal {L}}}}(\hat{u}_\ell )$ and $A\cap {{{\mathcal {L}}}}(\hat{u}_r)$ each have a non-empty intersection with exactly one of *R* and *B*, and they cannot intersect *W* because otherwise $A\cap {{{\mathcal {L}}}}(\hat{u})$ is a tricolored non-top component of ${\mathcal {P}}^{(1)}$, and then ${\mathcal {P}}^{(2)}$ would also have a tricolored non-top component by the definition of Make-Splittable, contradicting that $t=0$.
- Because $A\subseteq A_0$ was the top component at the moment Make-$(R\cup B)$ -compatible subdivided *A* into $A\cap {{{\mathcal {L}}}}(\hat{u})$ and $A\setminus {{{\mathcal {L}}}}(\hat{u})$, $A\setminus {{{\mathcal {L}}}}(\hat{u})$ is the top component in ${\mathcal {P}}^{(1)}$. By Lemma 13, $A\setminus {{{\mathcal {L}}}}(\hat{u})$ intersects $R\cup B$. By the conditions of Case 1 and property 5 in Lemma 4, it contains a node $x_W$ that is not a descendant of ${{\,\mathrm{lca}\,}}_2(A_0\cap (R\cup B))$. Finally, $A\setminus {{{\mathcal {L}}}}(\hat{u})$ is the only component in ${\mathcal {P}}^{(1)}$ that can cover a node on the path in $T_2$ from $\hat{u}$ to ${{\,\mathrm{lca}\,}}_2(A_0)$ (by the fact that $A_0$ was the unique top component in ${\mathcal {P}}^{(0)}$ and $A\setminus {{{\mathcal {L}}}}(\hat{u})$ is the unique top component in ${\mathcal {P}}^{(1)}$).

**Fig. 7:**
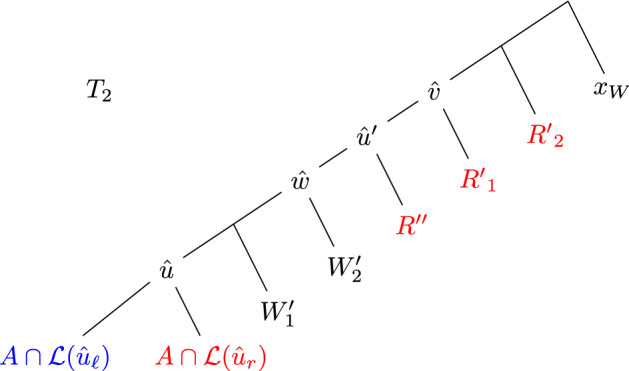
Illustration of the last part of the proof of Proposition 15. The sets $W'$ and $R'$ described in the proof are implicitly shown in the figure: $W=W_1'\cup W_2'$ and $R'=R_1'\cup R_2'$

Using the above, we now show we can find two components in ${\mathcal {P}}^{(3)}$ that are subsets of *A* and that satisfy condition (b) or (c) of Lemma 16. As argued above, $A\cap {{{\mathcal {L}}}}(\hat{u}_\ell ) \subseteq B$ and $A\cap {{{\mathcal {L}}}}(\hat{u}_r) \subseteq R$, so $A\cap {{{\mathcal {L}}}}(\hat{u})$ is splittable; Split will subdivide $A\cap {{{\mathcal {L}}}}(\hat{u})$ into a blue component $A\cap {{{\mathcal {L}}}}(\hat{u}_\ell )$ and a red component $A\cap {{{\mathcal {L}}}}(\hat{u}_r)$. There are a few cases to consider (illustrated in Fig. 7).

1. If there is no node on the path in $T_2$ from $\hat{u}$ to ${{\,\mathrm{lca}\,}}_2(A_0)$ that is covered by a red or blue component in ${\mathcal {P}}^{(3)}$, then we are done because $\hat{u}$ with $A\cap {{{\mathcal {L}}}}(\hat{u}_\ell )$ and $A\cap {{{\mathcal {L}}}}(\hat{u}_r)$ satisfy (c).
2. If there are nodes on the path from $\hat{u}$ to ${{\,\mathrm{lca}\,}}_2(A_0)$ that are covered by red or blue components, let $\hat{v}$ be the node closest to $\hat{u}$ for which this is the case. Suppose without loss of generality that $\hat{v}\in V_2[R']$ for some red component $R'$. We will show that there is another red component that can reach $\hat{v}$, so these two components and $\hat{v}$ satisfy condition (b).
  - If the nodes between $\hat{u}$ and $\hat{v}$ are not covered by any components in ${\mathcal {P}}^{(3)}$, then the red component $A\cap {{{\mathcal {L}}}}(\hat{u}_r)$ can reach $\hat{v}$.
  - Otherwise, let $\hat{w}$ be the node closest to $\hat{v}$ on the path from $\hat{u}$ to $\hat{v}$ that is covered by a component in ${\mathcal {P}}^{(3)}$. By definition of $\hat{v}$, this component is white, say $W'\in {\mathcal {P}}^{(3)}$. We claim (and prove below) that in Make-Splittable a node $\hat{u}'$ must have been chosen that created a non-top component $A'=W'\cup R''$, which was subsequently split into the white component $W'$ and red component $R''$ to obtain ${\mathcal {P}}^{(3)}$. By property 4 in Lemma 4, $\hat{u}'$ is not covered by $R''$ nor $W'$, so $R''$ and $W'$ are the leaves in $A'\cap {{{\mathcal {L}}}}(\hat{u}_r')$ and $A'\cap {{{\mathcal {L}}}}(\hat{u}_\ell ')$, with $\hat{u}_r', \hat{u}_\ell '$ being the two children of $\hat{u}'$. Hence, $R''$ can reach $\hat{u}'$. On the other hand, $\hat{w}$ is covered by $W'$, and thus $\hat{w}\prec \hat{u}'\prec \hat{v}$. By definition of $\hat{w}$, the nodes on the path from $\hat{u}'$ to $\hat{v}$ are not covered by any component in ${\mathcal {P}}^{(3)}$, and thus $R''$ can also reach $\hat{v}$.

It remains to prove that in 2(b), Make-Splittable selected a node $\hat{u}'$ that created a non-top component $W'\cup R''\subset {{{\mathcal {L}}}}(\hat{u}')$, which was subsequently split into $W', R''$ to obtain ${\mathcal {P}}^{(3)}$. First, observe that $W'$ and $R'$ were part of the top component in ${\mathcal {P}}^{(1)}$ (because they cover nodes on the path from $\hat{u}$ to ${{\,\mathrm{lca}\,}}_2(A_0)$). They cannot both have been part of the top component of ${\mathcal {P}}^{(2)}$, because by the conditions of Case 1 and property 5 of Lemma 4, the top component of ${\mathcal {P}}^{(2)}$ contains a white leaf $x_W$ that is not a descendant of ${{\,\mathrm{lca}\,}}_2(A_0\cap (R\cup B))$, and thus $W'\cup \{x_W\}$ covers all nodes on the path from $\hat{w}$ to ${{\,\mathrm{lca}\,}}_2(A_0\cap (R\cup B))$, which includes $\hat{v}$. So $R'$ and $W'\cup \{x_W\}$ overlap and cannot be in the same component of the splittable partition ${\mathcal {P}}^{(2)}$. Thus, Make-Splittable must have selected some $\hat{u}'$ when $W'$ and $R'$ became part of different components. Note that $W'$ became part of a non-top component. It remains to show this component contains no blue leaves. Note that otherwise such a blue leaf $x_B{}$, and a red leaf $x_R{}\in R'\cap {{{\mathcal {L}}}}(\hat{v})$ and a red leaf $y_R{}\in R'\setminus {{{\mathcal {L}}}}(\hat{v})$ (which exists because $\hat{v}$ is the lowest node on the path from $\hat{u}$ to ${{\,\mathrm{lca}\,}}_2(A_0)$ covered by $R'$) would all belong to the component $A\setminus {{{\mathcal {L}}}}(\hat{u})\in {\mathcal {P}}^{(1)}$, but since ${{\,\mathrm{lca}\,}}_2(x_B{},x_R{}) = \hat{v} \prec {{\,\mathrm{lca}\,}}_2(x_B{}, x_R{}, y_R{})$, this triple would be incompatible , contradicting that ${\mathcal {P}}^{(1)}$ is $(R\cup B)$-compatible . $\square$

#### Theorem 17

The Red-Blue Algorithm is a 2-approximation for the maximum agreement forest (MAF) problem.

#### Proof

By Theorem 8, the Red-Blue Algorithm returns a feasible solution to MAF. We showed how to construct a feasible solution for the dual linear program (D’); by Propositions 14 and 15, the objective value of the solution to MAF returned by the Red-Blue Algorithm is at most twice the objective value of this dual solution. The approximation guarantee follows by linear programming duality. $\square$

## A compact formulation of the LP

Here we give a compact formulation for (LP). This shows that it can be optimized efficiently. While this is not needed in our algorithm, it is possible that an LP-rounding based algorithm could achieve a better approximation guarantee, in which case this formulation will be of use. Moreover, the compact linear program explicitly encodes the structure of compatible sets in a way that (LP) does not; we believe this may provide additional structural insights in the future.

We remark that (LP) can also be shown to be polynomially solvable by providing a separation oracle for the dual. The dual of (LP) is similar to (D$'$), the dual of (LP$'$), except that *z* is indexed only by singletons and not arbitrary subsets of ${{{\mathcal {L}}}}$. This dual has a polynomial number of variables, but an exponential number of constraints. By the equivalence of separation and optimization, it suffices to provide a separation oracle for this dual. In particular, it suffices to solve the problem of finding a most violated constraint amongst

$$\begin{aligned} \sum _{v \in V[L] \setminus {{{\mathcal {L}}}}} y_v + \sum _{v \in L} z_v \le 1 \qquad \forall L \in {{{\mathcal {C}}}}, \end{aligned}$$

for some given *y* and *z*. If we relabel $z_v$ to $y_v$, making *y* a vector indexed by *V*, we can restate this as follows. Given some (positive or negative) weights *y* on the nodes of *V*, find a compatible subset *L* which maximizes $\sum _{v \in V[L]} y_v$. This is a weighted variant of the *maximum agreement subtree problem*; in other words, the maximum agreement subtree problem is the problem where $y_v=1$ for all $v\in {{{\mathcal {L}}}}$. Similar to the usual (unweighted) version [26], this can be solved in polynomial time via dynamic programming.

Assume for convenience that ${{{\mathcal {L}}}}= \{1,2,\ldots , n\}$. We will deviate from the notational conventions in the previous sections, and use *i* and *j* to denote leaves, and $t\in \{1,2\}$ to index the two input trees.

Let $Z$ denote the set of all pairs $(i_1,i_2) \in {{{\mathcal {L}}}}^2$ for which $i_1 \le i_2$. Consider a compatible set $L \subseteq {{{\mathcal {L}}}}$. For $t \in \{1,2\}$, we will use $T_t[L]$ to denote the subtree of $T_t$ on $V_t[L]$. Compatibility implies that $T_1[L]$ and $T_2[L]$ are isomorphic. What we will now do is represent the structure of these isomorphic trees by an out-arborescence *F*(*L*), where the nodes of the arborescence are elements of *Z*, and more precisely, are a subset of $\{ (i,j) : i,j \in L, i \le j \}$. We do this as follows.

- If *L* contains only a single element *i*, then *F*(*L*) is the arborescence consisting of the single vertex (*i*, *i*).
- Otherwise, let $L_1$ and $L_2$ be the partition of *L* into the leaves below the two children of the root of $T_1[L]$. Take $i_1$ be the smallest element of $L_1$ and $i_2$ the smallest element of $L_2$; we assume $i_1 < i_2$ (otherwise, swap $L_1$ and $L_2$). The root of *F*(*L*) will be chosen as $r := (i_1, i_2)$. Now recursively apply this procedure to $L_1$ and $L_2$, yielding arborescences $F(L_1)$ and $F(L_2)$; let $r_1$ and $r_2$ denote their respective roots. Note that $F(L_1)$ and $F(L_2)$ are necessarily disjoint, since $L_1$ and $L_2$ are disjoint. Then *F*(*L*) is defined to be the union of $F(L_1)$ and $F(L_2)$, along with the arcs $(r,r_1)$ and $(r,r_2)$.
  Observe that the pair $r_1$ is of the form $(i_1, i'_1)$ for some $i'_1$, since $i_1$ remains the smallest element of $L_1$, whereas $r_2$ is of the form $(i_2, i_2')$ for some $i_2'$. We call $(r,r_1)$ the *left* arc and $(r,r_2)$ the *right* arc leaving *r*.

So put differently, this procedure takes the tree $T_1[L]$ (or $T_2[L]$; it makes no difference), contracts all nodes with only a single child, orients all edges away from the root, and then assigns a label to each node. This label consists of a pair of leaves in *L*, chosen minimally amongst the leaves in each of the two subtrees below the node (aside from leaves, which are labelled by repeating the leaf twice). We also note that if *L* is not a compatible set, then we could still apply this procedure, but it would return different results when applied to $T_2[L]$ instead of $T_1[L]$.

With this representation of a compatible set in mind, we now construct a certain directed graph *D* on the vertex set $Z$. It essentially contains all possible arcs that could appear in an arborescence constructed from a compatible set. We will use $U_1$ for arcs that can appear as left arcs, and $U_2$ for arcs that can appear as right arcs: the arc set of *D* is $U_1\cup U_2$. With a slight abuse of notation, define ${{\,\mathrm{lca}\,}}_t(r) = {{\,\mathrm{lca}\,}}_t(i_1, i_2)$ for any $r=(i_1,i_2)\in Z$; we can think of ${{\,\mathrm{lca}\,}}_t(r)$ as being the node in $T_t$ that the pair *r* identifies. Given two nodes $r = (i_1, i_2)$ and $s = (j_1, j_2)$ in $Z$:

- $(r,s) \in U_1$ if ${{\,\mathrm{lca}\,}}_t(s) \prec {{\,\mathrm{lca}\,}}_t(r)$ for all $t\in \{1,2\}$ and $i_1 = j_1$;
- $(r,s) \in U_2$ if ${{\,\mathrm{lca}\,}}_t(s) \prec {{\,\mathrm{lca}\,}}_t(r)$ for all $t\in \{1,2\}$ and $i_2 = j_1$.

For any $L \subseteq {{{\mathcal {L}}}}$, define $Z_L = \{ (i,i) : i \in L\}$; these are the set of pairs in $Z$ that appear as labels for the leaves *L*. Let ${\mathcal {F}}$ denote the set of out-arborescences in *D* with leaf set contained in $Z_{{{\mathcal {L}}}}$ and where each internal node has one outgoing arc in $U_1$ and one outgoing arc in $U_2$. Then the above discussion implies that $L \in {{{\mathcal {C}}}}$ if and only if there is an $F(L) \in {\mathcal {F}}$ with leaf set $Z_L$. Let $\chi _F \in \{0,1\}^{U_1\cup U_2}$ be the characteristic vector of the arc set of *F*, for any $F \in {\mathcal {F}}$. Let $C_{{\mathcal {F}}}$ denote the cone generated by $\{\chi _F : F \in {\mathcal {F}}\}$, i.e., $y\in C_{{\mathcal {F}}}$ if and only if there exists $x \in {\mathbb {R}}^{{\mathcal {C}}}$ with $x \ge 0$ such that $y=\sum _{L \in {{{\mathcal {C}}}}: |L| \ge 2} x_L \chi _{F(L)}$.

We begin by giving a description of $C_{{\mathcal {F}}}$. For $r \in Z$, let $\delta ^+(r)$ denote the arcs in *D* leaving *r*, and $\delta ^-(r)$ the arcs entering *r*. For $S \subseteq U_1\cup U_2$, let $y(S) = \sum _{a \in S} y_a$.

### Lemma 18

 

$$\begin{aligned} C_{{\mathcal {F}}}= \bigl \{\; y \in {\mathbb {R}}_+^{U_1\cup U_2} : y(\delta ^+(r) \cap U_1)&= y(\delta ^+(r) \cap U_2)&\forall r \in Z\setminus Z_{{{\mathcal {L}}}}\\ y(\delta ^+(r) \cap U_1)&\ge y(\delta ^-(r))&\forall r \in Z\setminus Z_{{{\mathcal {L}}}}\; \bigr \}. \end{aligned}$$

### Proof

Let *Y* denote the cone described by the right hand side of the claimed equality. First, we observe that $Y \supseteq C_{{\mathcal {F}}}$. Consider any $F \in {\mathcal {F}}$. Then for any $r \in F\cap Z\setminus Z_{{{\mathcal {L}}}}$, *F* has precisely one arc entering *r*, precisely one arc leaving *r* that is in $U_1$, and one arc leaving *r* that is in $U_2$. Hence $\chi _F \in Y$, and therefore any conic combination of the $\chi _F$’s is also in *Y*.

It remains to show that $Y \subseteq C_{{\mathcal {F}}}$. Suppose $y \in Y$; we prove that $y \in C_{{\mathcal {F}}}$, proceeding by induction on the number of nonzero elements of *y*. The claim trivially holds if $y=0$, since $C_{{\mathcal {F}}}$ is a cone. So suppose $y\ne 0$.

We first claim that for any $r \in Z$ for which either $r \in Z_{{{\mathcal {L}}}}$ or $y(\delta ^+(r)) > 0$, there exists an arborescence $F \in {\mathcal {F}}$ rooted at *r* and contained in the *support* of *y*, by which we mean the set of arcs in *D* for which *y* is nonzero. To prove this, we can proceed by induction on $|{{\,\mathrm{lca}\,}}_1(r)|$. The claim is trivial if $|{{\,\mathrm{lca}\,}}_1(r)| = 1$, since then $r \in Z_{{{\mathcal {L}}}}$ and we take an arborescence consisting only of the node *r*. Otherwise, choose any $(r,r_1) \in U_1\cap \delta ^+(r)$ and $(r, r_2) \in U_2\cap \delta ^+(r)$ that are both in the support of *y*. Notice that one of them must exist because $y(\delta ^+(r)) > 0$, and then the other must exist as well, because of the equality in the definition of $C_{{\mathcal {F}}}$. As a result, $y(\delta ^-(r_1)) > 0$, and so the second constraint in the definition of *Y* implies that either $r_1\in Z_{{{\mathcal {L}}}}$ or $y(\delta ^+(r_1)) > 0$; the same holds for $r_{2}$. Hence by induction, we obtain arborescences $F_1$ and $F_2$ in the support of *y* rooted at $r_1$ and $r_2$ respectively. We have already noted that there is no node that both $r_1$ and $r_2$ can reach; thus $F_1$ and $F_2$ are disjoint. We obtain *F* by combining $F_1$, $F_2$ and the arcs from *r*.

Now choose $r=(i_1,i_2) \in Z$ such that $y(\delta ^-(r)) = 0$ but $y(\delta ^+(r)) > 0$ (such an *r* clearly exists, since *D* is acyclic and $y \ne 0$). By the above, we can find an arborescence $F \in {\mathcal {F}}$ rooted at *r* and contained in the support of *y*, Now set $y'= y - \epsilon \chi _F$, where $\epsilon$ is chosen maximally so that $y' \ge 0$. For every node *s* contained in *F* that is distinct from *r* and not in $Z_{{{\mathcal {L}}}}$, $\chi _F(\delta ^-(s)) = \chi _F(\delta ^+(s) \cap U_1) = \chi _F(\delta ^+(s) \cap U_2) = 1$. Further, $\chi _F(\delta ^-(r) = 0 < \chi _F(\delta ^+(r) \cap U_1) = \chi _F(\delta ^+(r) \cap U_2)$. It follows that $y' \in Y$. The choice of $\epsilon$ ensures that $y'$ has strictly smaller support than *y*, and so by induction, we deduce that $y' \in C_{{\mathcal {F}}}$. Hence $y = y' + \epsilon \chi _F$ is too. $\square$

Using Lemma 18, we now describe our compact formulation that we baptize $\text {LP}^\star$. For $t \in \{1,2\}$ and $v \in V_t$, let ${{\,\mathrm{lca}\,}}^{-1}(v) = \{ r \in Z: {{\,\mathrm{lca}\,}}_t(r) = v \}$, i.e., the set of all pairs of leaves with one leaf in *v*’s left subtree, and the other in its right subtree. 
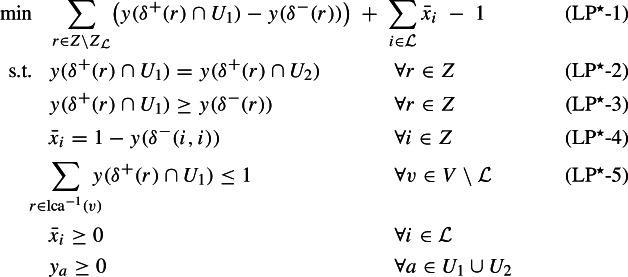


### Lemma 19

($\text {LP}^\star$) is equivalent to (LP).

### Proof

We begin by showing the “easy” direction, that a feasible solution *x* to (LP) can be converted to a feasible solution $(y,{\bar{x}})$ to ($\text {LP}^\star$) with the same objective value. Set ${\bar{x}}_i = x_{\{i\}}$ for all $i \in {{{\mathcal {L}}}}$, and $y = \sum _{L \in {{{\mathcal {C}}}}: |L| \ge 2} x_L \chi _{F(L)}$. By the “easy” direction of Lemma 18 (and the equality $\sum _{L \in {{{\mathcal {C}}}}: i \in L} x_L = 1$ for all $i \in {{{\mathcal {L}}}}$), we can deduce that $(y,{\bar{x}})$ is feasible to ($\text {LP}^\star$). Further, the objective values match: for any $L \in {{{\mathcal {C}}}}$ with $|L| \ge 2$, the contribution of the term $x_L \chi _{F(L)}$ in *y* to the objective value is exactly $x_L$ (only the root of *F*(*L*) contributes), and the term $\sum _{i \in {{{\mathcal {L}}}}} {\bar{x}}_i$ captures the fractional value of singleton components.

The “hard” direction, that a feasible solution $(y, {\bar{x}})$ to ($\text {LP}^\star$) can be converted to a feasible solution *x* to (LP) of the same objective value, follows in exactly the same way, but using the “hard” direction of Lemma 18. The constraints ($\text {LP}^\star$-2) and ($\text {LP}^\star$-3) ensure that $y\in C_{{\mathcal {F}}}$, and thus we can expand $y = \sum _{L \in {{{\mathcal {C}}}}: |L| \ge 2} x_L \chi _{F(L)}$ for some $x \ge 0$. Extending this *x* to singleton sets by defining $x_{\{i\}} = {\bar{x}}_i$ for all $i \in {{{\mathcal {L}}}}$ yields the desired solution to (LP). $\square$

## Conclusion

We have described a factor-2 approximation algorithm for the MAF problem with a quadratic running time. Unlike previous algorithms for the problem, we crucially exploit the power of linear programming duality in our analysis. A number of clear directions remain for future work.

**Fig. 8:**
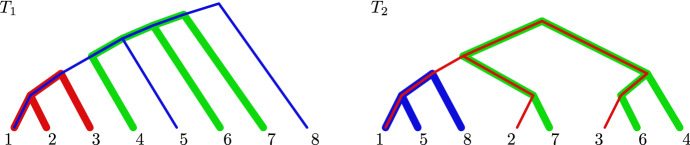
Example with integrality gap of $\tfrac{5}{4}$ for the new LP introduced in Section 4. An optimal solution to the LP-relaxation is indicated by the colors: the compatible sets corresponding to each of the components $\{1,2,3\}, \{1,5,8\}$ and $\{4,6,7\}$ (indicated by the colors red, blue and green respectively) have an *x*-value of $\tfrac{1}{2}$, as well as every singleton leaf set, except leaf 1; all other *x*-values are 0. The objective value of this solution is $\tfrac{1}{2}(3+7)-1 = 4$. An optimal solution to the ILP has 6 components, i.e., an objective value of 5. (One such optimal solution has the component $\{1,2,3\}$ along with singleton components)

Most obviously, is the question of whether the approximation factor can be further improved. The approximation ratio of our algorithm implies an upper bound of 2 on the integrality gap of our linear program. However, the largest lower bound on the integrality gap of our linear program that we are aware of is 5/4; Fig. 8 in Appendix 2 shows one of many examples achieving this bound. Despite extensive computational experiments on instances with a small number of leaves, we have not been able to find any examples with an integrality gap larger than 5/4. It is thus possible that our formulation could be used as the basis for an improved algorithm (though we would expect such an algorithm to be quite different from the algorithm presented here).

One natural idea would be to apply another powerful and successful technique in the theory of approximation algorithms, namely *LP rounding*. We have shown that the LP relaxation can be efficiently optimized via an equivalent compact formulation. It should, however, be noted that such an approach will be much slower than the purely combinatorial algorithm presented here, where the ILP formulation is used only in the analysis. It may thus not be the most promising approach for an algorithm of practical relevance.

Our ILP formulation may also be useful for *exactly* solving the MAF problem. Although it is NP-hard, ILP solvers are very successful in practice. Since our formulation appears to be quite strong, it may work better in practice than simpler formulations, such as the one of Wu.

Aside from improving the approximation factor, another natural avenue to pursue is improving the running time. A factor-2 approximation algorithm with a linear or near-linear running time, to match what has been achieved with a factor-3 approximation, would clearly be very desirable. It does not seem straightforward to improve the running time of our current algorithm; quite substantial changes would likely be needed.

Another very natural direction is to consider other variants of the MAF problem. For instance, the variation with more than two trees, where the current best approximation factor is 3 [7]; or the generalization to non-binary trees. It is straightforward to extend our formulation to both of these setting.

Finally, it must be admitted that our algorithm, and especially its analysis, is far from simple. A truly *simple* algorithm for MAF with an approximation factor of 2, if one can be found, will certainly require understanding its structure even more deeply.

## Appendix A The running time

It is quite clear from the definition of the Red-Blue Algorithm that it runs in polynomial time. In this section we show that it can be implementated to run in $O(n^2)$ time, where *n* denotes the number of leaves. (We work in the random access machine model of computation, and assume a word size of $\Omega (\log n)$.)

We note that our presentation is focused on showing the bound on the running time as straightforwardly as possible, and there are some places where a more careful implementation is more efficient. However, we have not been able to find an implementation with an overall running time of $o(n^2)$.

We assume ${\mathcal {P}}$ is a given partition (not overlapping in $V_2$), that is stored such that we can query the size of any component in constant time, and that for each node $\hat{u}\in V_2$, we can query $A_{\hat{u}}$, the component in ${\mathcal {P}}$ that covers $\hat{u}$ (which will be equal to $\emptyset$ if ${\mathcal {P}}$ does not cover $\hat{u}$), and $s(\hat{u}) = |{{{\mathcal {L}}}}(\hat{u})\cap A_{\hat{u}}|$. Note that we can determine this information by a bottom-up pass of $T_2$ in *O*(*n*) time. We will recompute it whenever we refine ${\mathcal {P}}$; since there can only be at most $n-1$ refinement operations, the total time to maintain this information is $O(n^2)$.

By Harel [14] (see also [2, 15]), we furthermore may assume that the computation of ${{\,\mathrm{lca}\,}}_i(u,v)$ for given nodes $u,v\in V_i$ takes constant time (after a linear preprocessing time). It immediately follows from this that we can determine whether or not $u \preceq v$ in tree $T_i$ in constant time as well.

We will show that the time between subsequent refinements of ${\mathcal {P}}$ is *O*(*n*). This bounds the time of the main loop of the algorithm by $O(n^2)$. The only remaining part of the algorithm is the Merge-Components step, which will perform at most $n-1$ merges, each of which can clearly be done in *O*(*n*) time.

### Finding a lowest root of infeasibility

We make a single pass through $T_1$, in bottom-up order (starting from the leaves), until we find a root of infeasibility . We will spend constant time per node, thus showing that the time to find a lowest root of infeasibility is *O*(*n*).

For each node $u\in V_1$ that we have already considered, $A_u$ references the component $A \in {\mathcal {P}}$ which covers *u*, with $A_u = \emptyset$ if there are no such components. (If there are multiple such components, *u* is a root of infeasibility.) Furthermore, $\hat{p}_{u}$ is equal to ${{\,\mathrm{lca}\,}}_2(A_u \cap {{{\mathcal {L}}}}(u))$, and $s(u)$ is the size of $A_u \cap {{{\mathcal {L}}}}(u)$. Observe that for any $x \in {{{\mathcal {L}}}}$, we know $A_x$, the component containing *x*, and $s(x) = 1$ and $\hat{p}_{x} = x$.

Given a non-leaf node $u \in V_1$, with children $u_1$ and $u_2$ that have already been considered, we can determine whether *u* is a root of infeasibility , and, if not, determine $A_u, \hat{p}_{u}$ and $s(u)$, in constant time: If either (or both) of $A_{u_1}$ and $A_{u_2}$ do not cover *u* (which can be determined by checking if $A_{u_i} = \emptyset$ or $s(u_i) = |A_{u_i}|$), set all the values according to which child (if any) does cover *u*, and end the consideration of node *u*. So assume from now on that both do cover *u*.

If $A_{u_1} \ne A_{u_2}$, then *u* satisfies the second condition of a root of infeasibility , and we are done. Otherwise, $A_u = A_{u_1} = A_{u_2}$. Set $\hat{p}_{u} = {{\,\mathrm{lca}\,}}_2(\hat{p}_{u_1}, \hat{p}_{u_2})$ and $s(u) = s(u_1) + s(u_2)$. If $\hat{p}_{u_1} \nprec \hat{p}_{u}$ or $\hat{p}_{u_2} \nprec \hat{p}_{u}$, then ${{{\mathcal {L}}}}(u)$ is incompatible, and *u* satisfies the first condition of a root of infeasibility . If $s(\hat{p}_{u}) = |A_u|$ and $s(u) < |A_u|$ then *u* satisfies the third condition for being a root of infeasibility : by $s(u) < |A_u|$, we know $A_u\setminus {{{\mathcal {L}}}}(u)\ne \emptyset$. For any $w\in A_u\setminus {{{\mathcal {L}}}}(u)$, ${{\,\mathrm{lca}\,}}_1( A_u \cap {{{\mathcal {L}}}}(u) ) \preceq u \preceq {{\,\mathrm{lca}\,}}_1( A_u \cap {{{\mathcal {L}}}}(u) \cup \{ w \} )$, while by $s(\hat{p}_{u})=|A_u|$ we know that ${{\,\mathrm{lca}\,}}_2( A_u \cap {{{\mathcal {L}}}}(u) ) = {{\,\mathrm{lca}\,}}_2( A_u )$. So $A_u\cap {{{\mathcal {L}}}}(u)\cup \{w\}$ is incompatible for any $w\in A_u\setminus {{{\mathcal {L}}}}(u)$. Otherwise, *u* is not a root of infeasibility , and we finish our consideration of *u*.

Once we have determined the coloring (*R*, *B*, *W*), we compute $|A\cap C|$ for each component $A\in {\mathcal {P}}$ and $C\in \{R, B, W\}$. We also compute three additional labels for each node $\hat{u}\in V_2$: $s_C(\hat{u}) = |A_{\hat{u}} \cap {{{\mathcal {L}}}}(\hat{u})\cap C|$ for $C\in \{R, B, W\}$. This information can be determined by a bottom-up traversal of $T_2$ in *O*(*n*) time. We assume this information is updated whenever the partition is refined.

#### Make-$(R\cup B)$-compatible

Consider the nodes of $T_2$ in bottom-up order, until we find a node $\hat{u}$ such that both $s_B(\hat{u})>1$ and $s_R(\hat{u})>1$. Since $\hat{u}$ is a lowest such node, if $|A_{\hat{u}}\cap R| = s_R(\hat{u})$ and $|A_{\hat{u}}\cap B|= s_B(\hat{u})$, then $A_{\hat{u}}$ is $(R\cup B)$-compatible ; otherwise $\hat{u}$ is precisely as indicated in Make-$(R\cup B)$ -compatible.

#### Make-splittable

We again consider the nodes in $T_2$ in bottom-up order. For any node $\hat{u}$ with $A_{\hat{u}} \ne \emptyset$, using $s_C(\hat{u})$ for $C\in \{R, B, W\}$, we can check in *O*(1) time whether $A_{\hat{u}} \cap {{{\mathcal {L}}}}(\hat{u})$ is bicolored, and that for any $C \in \{R,B,W\}$ with $A_{\hat{u}} \cap C \ne \emptyset$, that $s_C(\hat{u}) < |A_{\hat{u}} \cap C|$ (and hence $(A_{\hat{u}} \setminus {{{\mathcal {L}}}}(\hat{u})) \cap C \ne \emptyset$).

#### Split

Note that a regular split of a component *A* can be done in *O*(*n*) time, by simply checking the color of each leaf in *A* and partitioning *A* accordingly. We now show how to check if *A* needs a Special-Split (and if so which of the two possible refinements is applied) by considering the nodes in $V_2[A]$ in bottom-up order.

If *A* is tricolored , then the fact that *A* is $(R\cup B)$-compatible and splittable implies that there exist $\hat{u}_R$ and $\hat{u}_B$ that are covered by *A* and for which $A\cap {{{\mathcal {L}}}}(\hat{u}_R)= A\cap R$ and $A\cap {{{\mathcal {L}}}}(\hat{u}_B)=A\cap B$. Using a bottom-up traversal of $V_2$ will find $\hat{u}_R$ and $\hat{u}_B$ (they are the first nodes $\hat{v}$ encountered such that $A_{\hat{v}}=A$ and $s_C(\hat{v})=|A\cap C|$ for $C=R$ and *B* respectively).

Given $\hat{u}_R$ and $\hat{u}_B$, we check if a Special-Split is required, by considering $\hat{u} = {{\,\mathrm{lca}\,}}_2(A\cap (R\cup B))={{\,\mathrm{lca}\,}}_2(\hat{u}_R,\hat{u}_B)$; a Special-Split is required exactly if $s({{\,\mathrm{lca}\,}}_2(\hat{u}_R,\hat{u}_B))<|A|$, since in that case any $x_W\in A\setminus {{{\mathcal {L}}}}({{\,\mathrm{lca}\,}}_2(\hat{u}_R,\hat{u}_B))$ forms a compatible triple with any $x_R\in A\cap R, x_B\in A\cap B$. If, in addition, $s_W({{\,\mathrm{lca}\,}}_2(\hat{u}_R,\hat{u}_B))=0$, we know that every tricolored triple in *A* is compatible.

#### Find-merge-pair

We need to determine if there exist two components (both intersecting $R \cup B$) that can be merged, in time *O*(*n*). If such components are found, then we can take a non-white leaf in each component and add this pair to pairslist . Recall Lemma 16, which enumerates all possible situations where a potential merge may exist.

- ${\mathcal {P}}$ *has a* bicolored *component. * This component must have been created by an application of Special-Split, splitting some component $A \cup A^* \in {\mathcal {P}}^{(2)}$ into *A* and $A^*$. As discussed in the proof of Lemma 16, simply undoing this split is a valid merge. Since Special-Split is invoked at most once per iteration, we can simply add a pair to pairslist during Special-Split.
- *There is a node* ${{\hat{u}}}\in V_2$ *that can be reached by two red or two blue components that were part of the same component at the start of the current iteration.* By Lemma 12 (and property 1 of Lemma 4), any two components that are not white that were created in the current iteration must have been part of the same partition at the start of the iteration. We may assume that we can check for each component in constant time whether it was created in the current iteration.
  We work bottom-up in $T_2$, and set ${\mathcal {B}}_{\hat{u}}$ to be the set of red and blue components that were created in the current iteration, and that can reach $\hat{u}$ for every $\hat{u}\in V_2$. If ${\mathcal {B}}_{\hat{u}}$ contains two components of the same color, these two components can be merged, and we terminate.
  Note that if ${\mathcal {B}}_{\hat{u}}$ ever contains three components, we will have found a merge and the algorithm will terminate. This ensures that we can compute this for $\hat{u}$ in constant time, given the values of $A_{\hat{u}}$ and ${\mathcal {B}}_{\hat{u}_1}$ and ${\mathcal {B}}_{\hat{u}_2}$ for the children of $\hat{u}$.
- *There is a node* $\hat{u}\in V_2$ *that can be reached by a red and a blue component that were part of the same component* $A_0$ *at the start of the current iteration, but is not covered by these components. Furthermore, the node* $\hat{u}$ *must satisfy that the nodes on the path from* $\hat{u}$ *to* ${{\,\mathrm{lca}\,}}_2(A_0)$ *are not covered by any red or blue component.* Note that by Lemma 12, $A_0$ is the only component that was modified in the current iteration, so for this second condition we can simply check that no node on the path from $\hat{u}$ to the root of $V_2$ is covered by a red or blue component that was created in the current iteration.
  If we did not find two components of the same color that can be merged, we have found ${\mathcal {B}}_{\hat{u}}$ for every $\hat{u}\in V_2$, where $|{\mathcal {B}}_{\hat{u}}|\le 2$. We now work top-down in $T_2$. If we encounter a node $\hat{u}$ that is covered by a red or blue component that was created in the current iteration, we stop and do not consider the descendants of $\hat{u}$ (since for any such a descendant, $\hat{u}$ is on its path to ${{\,\mathrm{lca}\,}}_2(A_0)$). If we encounter a node $\hat{u}$ such that $|{\mathcal {B}}_{\hat{u}}|=2$ and $\hat{u}$ is not covered by any component, the two components in ${\mathcal {B}}_{\hat{u}}$ can be merged, and we may terminate.

## Appendix B Integrality gap lower bounds

We show a lower bound on the integrality gap of $\tfrac{16}{5}$ for the integer linear program formulation of Wu [31]. Recall that a solution to MAF can be viewed as the leaf sets of the trees in a forest, obtained by deleting edges from the input trees. The formulation has binary variables $x_e$ for every edge $e \in T_1$, indicating whether *e* is deleted from $T_1$. We use $P_1(i, j)$ to denote the set of edges in $T_1$ on the path between leaves *i* and *j*, and $P_2(i, j)$ to denote the set of edges in $T_2$ on the path between leaves *i* and *j*. Wu’s linear program [31] is given by:

$$\begin{aligned} {{\,\mathrm{minimize}\,}}\quad&\sum _e x_e \\ \text {s.t.}\quad&\sum _{e \in P_1( i,j ) \cup P_1( i,k ) \cup P_1( j,k ) } x_e \ge 1&\text{ for } \text{ all } \text{ incompatible } \text{ triples } i,j,k \\&\sum _{e \in P_1( i,j )} x_e + \sum _{e \in P_1( k,\ell )} x_e \ge 1&\mathop {\,}\limits _{\begin{array}{c} \hbox { for all two pairs }(i,j) \hbox { and }(k,\ell ) \hbox { for which } \\ P_1(i,j) \cap P_1(k,\ell ) = \emptyset , \hbox { and }P_2(i,j) \cap P_2(k,\ell ) \ne \emptyset \end{array}} \end{aligned}$$

The first family of constraints ensures that at least one edge of the paths between *i* and *j*, *i* and *k*, and *j* and *k* has to be deleted for each inconsistent triple *i*, *j* and *k*. The second family of constraints ensures that at least one edge is deleted for every pair of paths between *i* and *j*, and *k* and $\ell$ that are disjoint in $T_1$, but for which the corresponding paths in $T_2$ are not disjoint.

### Lemma 20

The integrality gap of the linear program of Wu [31] is at least $\tfrac{16}{5}$.

### Proof

Let $n=2^k$ for some *k* even. We label each internal node in both $T_1$ and $T_2$ with a binary string: the roots get the empty string as label, and given an internal node *u* its left child gets *u*’s label with a “0” appended, and its right child gets *u*’s label with a “1” appended. In $T_1$, the leaves are labelled in the same way as the internal nodes, with a binary string of length *k*. In $T_2$, the binary string is *reversed* to give the label of the leaf. For example, the leftmost leaf (of both trees) has label $00\cdots 0$, and the leaf to the right of it has label $0\cdots 01$ in $T_1$, and $10\cdots 0$ in $T_2$.

Consider the internal nodes whose labels are strings of length strictly less than *k*/2; there are exactly $2^{k/2}-1=\sqrt{n}-1$ such nodes in each tree. We claim that any component *A* must cover at least $|A|-1$ of these internal nodes. To see this, consider the set of internal nodes in $V_t$ that have two children in the subtree of $T_t$ induced by *A* for $t=1,2$. We will call such nodes *bifurcating*. Observe that there are $2(|A|-1)$ such nodes. Furthermore, since *A* is compatible , there is a 1-1 mapping *f* from the bifurcating nodes in $V_1$ to the bifurcating nodes in $V_2$, where, ${{{\mathcal {L}}}}(u)\cap A={{{\mathcal {L}}}}(f(u))\cap A$. Now, the label for a bifurcating node $u\in V_1$ is the maximum length prefix that the binary strings for the leaves in ${{{\mathcal {L}}}}(u)\cap A$ have in common, and the label for *f*(*u*) is the reverse of the maximum length suffix the leaves in ${{{\mathcal {L}}}}(u)\cap A$ have in common. Hence, at least one of *u* and *f*(*u*)’s labels has length less than *k*/2.

The fact that any component *A* must cover at least $|A|-1$ of the $2\sqrt{n}-2$ internal nodes with labels of length less than *k*/2 implies that any partition that does not overlap must have at least $n-2\sqrt{n}+2$ components. Thus the optimal value of the integer program is at least $n-2\sqrt{n}+1$.

On the other hand, the LP relaxation of the integer program has a feasible solution with objective value $\frac{5}{16}n$: set a value of $\tfrac{1}{4}$ on the edges to each leaf in the tree (i.e., from an internal node with a label of length $k-1$ to a node with a label length *k*), and a value of $\tfrac{1}{8}$ on all edges between nodes with labels of length $k-2$ to nodes with labels of length $k-1$. This implies a lower bound of $\lim _{n\rightarrow \infty }\frac{n-2\sqrt{n}+1}{\frac{5}{16}n}=\tfrac{16}{5}$ on the integrality gap. $\square$

As remarked in the introduction, the largest integrality gap for our formulation that we are aware of is 5/4. The instance is described in Fig. 8.
